# Design and analysis of LacI-repressed promoters and DNA-looping in a cyanobacterium

**DOI:** 10.1186/1754-1611-8-4

**Published:** 2014-01-27

**Authors:** Daniel Camsund, Thorsten Heidorn, Peter Lindblad

**Affiliations:** 1 Microbial Chemistry, Department of Chemistry–Ångström Laboratory, Science for Life Laboratory, Uppsala University, P.O. Box 523, SE-75120 Uppsala, Sweden; 2 Bioforsk, Frederik A Dahls vei 20, 1432 Ås Oslo, Norway

**Keywords:** Cyanobacteria, Lac repressor, Synthetic biology, Transcriptional systems, P*trc*, *Synechocystis*, LacI, Metabolic engineering tools, Transcriptional regulation

## Abstract

**Background:**

Cyanobacteria are solar-powered prokaryotes useful for sustainable production of valuable molecules, but orthogonal and regulated promoters are lacking. The Lac repressor (LacI) from *Escherichia coli* is a well-studied transcription factor that is orthogonal to cyanobacteria and represses transcription by binding a primary *lac* operator (*lacO*), blocking RNA-polymerase. Repression can be enhanced through DNA-looping, when a LacI-tetramer binds two spatially separated *lacO* and loops the DNA. P*trc* is a commonly used LacI-repressed promoter that is inefficiently repressed in the cyanobacterium *Synechocystis* PCC 6803. P*trc*2O, a version of P*trc* with two *lacO*, is more efficiently repressed, indicating DNA-looping. To investigate the inefficient repression of P*trc* and cyanobacterial DNA-looping, we designed a P*trc*-derived promoter library consisting of single *lacO* promoters, including a version of P*trc* with a stronger *lacO* (Ptrc1O-proximal), and dual *lacO* promoters with varying inter-*lacO* distances (the P*trc*2O-library).

**Results:**

We first characterized artificial constitutive promoters and used one for engineering a LacI*-*expressing strain of *Synechocystis*. Using this strain, we observed that P*trc*1O-proximal is similar to P*trc* in being inefficiently repressed. Further, the P*trc*2O-library displays a periodic repression pattern that remains for both non- and induced conditions and decreases with longer inter-*lacO* distances, in both *E. coli* and *Synechocystis*. Repression of P*trc*2O-library promoters with operators out of phase is less efficient in *Synechocystis* than in *E. coli*, whereas repression of promoters with *lacO* in phase is efficient even under induced conditions in *Synechocystis*. Two well-repressed P*trc*2O promoters were highly active when tested in absence of LacI in *Synechocystis*.

**Conclusions:**

The artificial constitutive promoters herein characterized can be utilized for expression in cyanobacteria, as demonstrated for LacI. The inefficient repression of P*trc* and P*trc*1O-proximal in *Synechocystis*, as compared to *E. coli*, may be due to insufficient LacI expression, or differences in RNAP subunits. DNA-looping works as a transcriptional regulation mechanism similarly as in *E. coli*. DNA-looping contributes strongly to P*trc*2O-library repression in *Synechocystis*, even though they contain the weakly-repressed primary *lacO* of P*trc*1O-proximal and relatively low levels of LacI/cell. Hence, *Synechocystis* RNAP may be more sensitive to DNA-looping than *E. coli* RNAP, and/or the chromatin torsion resistance could be lower. Two strong and highly repressed P*trc*2O promoters could be used without induction, or together with an unstable LacI.

## Background

Cyanobacteria, solar-powered prokaryotes that fix CO_2_ from air during photosynthesis, can be engineered to produce a variety of valuable molecules and fuels sustainably [1,2], and hence present a literally green alternative to the use of fossil fuels. The metabolic engineering efforts of even common biotechnological chassis such as *Escherichia coli (E. coli)*, has illustrated the need for synthetic biology to provide new tools, e.g. minimal chassis, vectors and genetic control systems [3]. This is all the more true for cyanobacteria, and even though tools are available for cyanobacterial synthetic biology, more tools, especially promoters, are needed [4,5]. Further, cellular context effects, cross-talk and interactions among genetic components and control systems are some of many often unpredictable causes for failure in engineered biological systems [6]. Orthogonal biological systems, which display minimal cross-talk because their components are decoupled from each other and preferably from the host, could help mitigate these problems [7]. However, most promoters previously used for engineering cyanobacteria are native to the chassis in question [8], so there is a specific lack of orthogonal promoters, constitutive as well as regulated.

**Figure 1:**
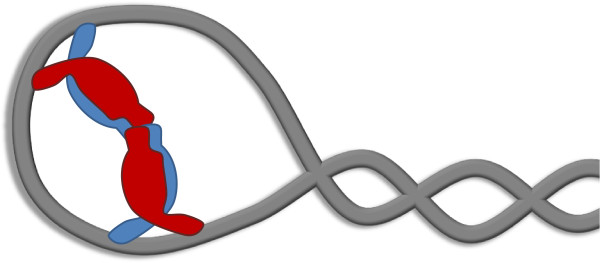
**Conceptual image of a Lac repressor tetramer binding inside of an apical loop of plectonemically supercoiled DNA.** Inspired by [20].

The Lac repressor (LacI) from *E. coli* is a well-studied tetrameric (a dimer of dimers) transcription factor [9] orthogonal to cyanobacteria that represents a model system and, in combination with an optimized promoter a tool, for engineering synthetic cyanobacterial transcriptional systems. In wild-type *E. coli*, LacI represses transcription of the *lacZYA* operon mainly by binding its primary *lac* operator (*lacO1*) that is located downstream of and overlapping with 1 bp the transcription start site (TSS) of the promoter [10]. Repression can be relieved by induction using lactose or its stable analog isopropyl β-D-1-thiogalactopyranoside (IPTG), which reduces the affinity of LacI towards its operator approximately 1000-fold [11]. The repression of the *E. coli lac* operon also depends on the auxiliary operators to which LacI binds with differing affinity. Of the three wild-type operators (the primary *lacO1* and the auxiliary operators *O2* and *O3*) that display an approximate dyad symmetry and an artificial perfectly symmetric *lacO (lacO*sym) [12], *lacO*sym was found to bind LacI with the greatest affinity, with the binding affinities ordered as *lacO*sym > *lacO1* > *lacO2* > *lacO3*[13]. Early on, it was found that the auxiliary *lac* operators, which are spatially separated from the primary *lacO1,* improve repression through a DNA-looping mechanism that increases the effective local concentration of LacI at the primary repression site [14]. A LacI tetramer bound to separately located *lacO* on the same side of the DNA-helix would be able to bend or loop the DNA (Figure 1). Vice versa, two *lacO* on different sides of the helix would not be able to simultaneously bind LacI by simply looping the DNA, making the formation of LacI-DNA-loops in geometrically unfavorable configurations more difficult. The DNA-looping model of cooperative tetrameric LacI-repression was confirmed in vitro [15], and in vivo [16]. Further, these and subsequent studies showed that DNA-loop-dependent repression varies periodically as a function of the distance between the two operators, with a period of about 11 bp or the approximate numbers of bp per helical turn of supercoiled chromatin DNA in vivo [17-22]. However, it should be noted that the helical repeat of in vivo chromatin DNA (referred to as DNA) will depend on the associated protein factors [20]. In a comprehensive in vivo DNA-looping study using a fixed primary *lacO1* for repression of a *lac*UV5 promoter, the upstream position of an auxiliary *lacO*sym was varied by varying the length of a spacer between the −35 box and the operator, effectively varying the inter-*lacO* distance from 57.5 to about 1500 bp [23]. While confirming previous results regarding DNA-loop mediated repression, this study also found that repression is phase-dependent from the shortest inter-*lacO* distance tested up to approximately 200 bp, that repression is more effective for shorter distances, and that phasing was not observed for distances above 400 bp. The strong repression phase-dependency for short inter-*lacO* distances and the lack of phase-dependency for longer distances was explained by the strong torsion resistance of short segments of DNA, making pure looping more energetically favorable than twisting, and the lower torsion resistance of longer stretches of DNA, removing the barrier for twisted loop formation.

We previously introduced and characterized two LacI-regulated promoters, P*trc*[24] and P*trc*2O, in the unicellular model cyanobacterium *Synechocystis* PCC 6803 (*Synechocystis*) [25]. Both promoters contain the same P*trc* core promoter, a chimera of the *E. coli trp* operon and *lac*UV5 promoters, including the proximal *lacO1* from the *lac* operon, and proved to be highly active in *Synechocystis* in the absence of LacI. This was expected since P*trc* contains the optimally spaced *E. coli* consensus −35 and −10 elements, which are similar to the consensus −35 and −10 boxes of the cyanobacterial group 1 promoters that are recognized by the primary sigma factor under normal growth conditions [26]. However, we observed that P*trc* is less efficiently repressed by heterologously expressed LacI in *Synechocystis* than in an *E. coli* strain over-expressing LacI. Further, P*trc*2O, a modified P*trc* with an additional *lacO*sym inserted further upstream of the −35 element to enable DNA-loop-enhanced repression, was more efficiently repressed in *Synechocystis*, but could not be efficiently induced [25]. The efficient repression of P*trc*2O, even under full IPTG induction, implies that insufficient levels of heterologously expressed LacI was not the reason for the inefficient repression of P*trc* in *Synechocystis*. In contrast to these results, P*trc* in combination with the *lacI*^*q*^ gene has proven to be both well-repressed and inducible in the cyanobacterium *Synechococcus elongatus* PCC 7942 [27]. However, when a recent study used a similar P*trc* and *lacI*^*q*^ setup in *Synechocystis*, no or very little repression of P*trc* could be detected, whereas the contrary was true in *E. coli*[28], in line with our previous results [25]. Further, P*tac*[29], which differs from P*trc* only in that the spacer between the −35 and −10 boxes is 16 bp instead of 17 bp [24], was only partially repressed by LacI when tested in the cyanobacterium *Nostoc* PCC 7120 [30]. The difference in repression of P*trc* between *E. coli* and *Synechocystis* could be due to differences in the cyanobacterial RNA polymerase (RNAP) complex [31,32] and in the interactions between RNAP, promoter and LacI, or simply because of differences in the amount of LacI per cell. Further, P*trc*2O could be efficiently repressed in *Synechocystis*, implying that DNA-looping through the LacI-tetramer may work in a similar way as in *E. coli*. While DNA curvature has been previously recognized to affect gene expression in cyanobacteria [33], as far as we are aware, gene regulation through DNA-looping has not previously been experimentally studied in cyanobacteria.

In the present study, we characterized minimal artificial promoters that can be utilized for stable constitutive expression in cyanobacteria, as demonstrated by using one of them to engineer a LacI-expressing strain of *Synechocystis*. Using this strain, we characterized a version of P*trc* with a stronger *lacO*, P*trc*1O-proximal, which similarly to P*trc* display limited repression as compared to its more efficient repression in *E. coli*. Possible reasons for this were found in the differences between *E. coli* and cyanobacterial RNA polymerases, or in the lower number of LacI/cell in the LacI-expressing strain of *Synechocystis* as compared with the LacI over-expressing strains of *E. coli* herein tested. Further, we characterized the P*trc*2O-library in both *E. coli* and *Synechocystis* and observe several similarities and differences between the two species. While the system displays a similar periodic repression pattern in both, illustrating the presence of DNA-looping in cyanobacteria, differences are found. These differences in repression and induction are attributed to the differing repression efficiency of the proximal *lac* operator of P*trc*1O-proximal, and potential differences between the RNAP sensitivity to DNA-looping and/or the in vivo chromatin DNA torsion-resistance between the two species. These findings will aid in the understanding of native cyanobacterial transcriptional systems and have design implications for engineering synthetic regulated promoters.

## Results

### Characterization of constitutive promoters in *Synechocystis*

**Figure 2:**
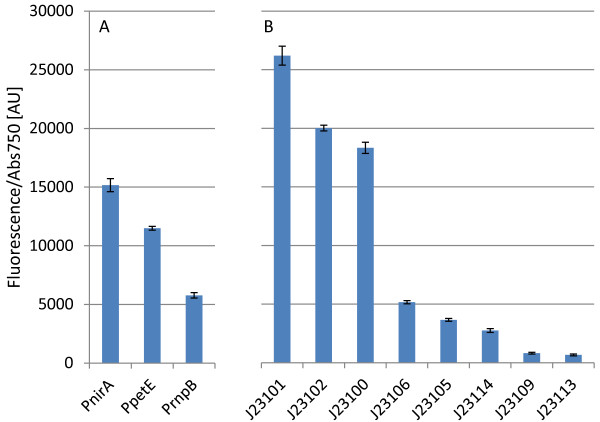
**Promoter activities per cell characterized using an EYFP reporter construct carried on pPMQAK1 in *Synechocystis *PCC 6803. A**. Native promoters from *Synechocystis* included for comparison. P*nirA* is the nitrate reductase promoter, P*petE* is the plastocyanin promoter and P*rnpB* is the RNase P subunit B promoter. **B**. Minimal constitutive promoters from an *Escherichia coli* synthetic promoter library. The J23### promoters correspond to the BBa_J23### BioBrick parts obtained from the iGEM Registry [34]. Promoter activities per cell are given in fluorescence/absorbance at 750 nm in arbitrary units [AU]. Error bars indicate standard error of the mean (n = 6).

To investigate the activity of minimal and constitutive orthogonal promoters in the unicellular cyanobacterium *Synechocystis* PCC 6803 (*Synechocystis*), eight members from an artificial promoter library spanning a wide range of activities in *Escherichia coli* were selected for testing using a pPMQAK1-carried [25] RBS* [4] EYFP fluorescent protein reporter construct. The artificial BioBrick promoters BBa_J23100, BBa_J23101, BBa_J23102, BBa_J23105, BBa_J23106, BBa_J23109, BBa_J23113 and BBa_J23114 [34] were compared to three promoters native to *Synechocystis*; the nitrate reductase promoter P*nirA,* the plastocyanin promoter P*petE* and the RNase P subunit B promoter P*rnpB*, which have been previously used for engineered expression, using the same reporter construct. The constitutive artificial promoters were found to span a wide range of activities in *Synechocystis*, encompassing and expanding the range covered by the three native promoters (Figure 2).

### Construction of a LacI-expressing and a Chloramphenicol resistance-only strain of *Synechocystis*

In our previous study [25] we found that LacI expressed from pPMQAK1 using P*rnpB* and the BBa_B0034 [34] ribosome binding site was sufficient for repression of the original P*trc*2O promoter. For constructing a strain of *Synechocystis* expressing similar levels of LacI from the chromosome instead of from the pPMQAK1 vector, we selected the minimal, constitutive and orthogonal BBa_J23114 promoter (weaker than the P*rnpB* promoter, as characterized in this study) in combination with the RBS* ribosome binding site that was found to be stronger than BBa_B0034 in *Synechocystis*[4]. Since the copy number of pPMQAK1 is expected to be similar to the copy number of the *Synechocystis* chromosome [25] or lower, taking newer data measuring the copy number of the chromosome to about 40 to 220 per cell depending on strain and growth phase into account [35], and assuming that the promoter activities are similar on the pPMQAK1 plasmid and the chromosome, we expected levels of LacI expression to be similar or higher than in our previous study. The LacI-expression cassette, consisting of a forward terminator blocking potentially incoming RNA polymerases from the native part of the chromosome, the BBa_J23114, RBS* and *lacI* CDS expression unit, and another forward terminator to stop BBa_J23114-promoted RNAP from reading out into the subsequent chloramphenicol resistance cassette, was inserted into a previously used neutral site of the chromosome using homologous recombination. The obtained LacI-enhanced strain of *Synechocystis* was confirmed to be fully segregated using PCR with primers flanking the insertion site, and further the areas around the insertion sites were sequence-verified using the same primers. LacI expression was confirmed through western blotting using LacI antibodies (data not shown) and confirmed functional through LacI repression and IPTG induction assays (see following results). Further, as known numbers of cells were used for the western blot of the LacI-enhanced strain of *Synechocystis* and the two *E. coli* strains used herein, NEB5α and DH5αZ1, we were able to quantify the relative levels of LacI/cell among the three strains. NEB5α has about 5X as many LacI/cell as compared to the LacI-enhanced strain of *Synechocystis*, and DH5αZ1 has about 16X as many LacI/cell. Using a previously published value for the number of LacI tetramers/cell in DH5αZ1, 3000/cell [36], as a standard, we estimated the number of LacI tetramers/cell in the LacI-enhanced strain of *Synechocystis* to be 190, and the number of LacI/cell in NEB5α to be about 880.

Finally, to enable the characterization of promoters in a control strain grown under the same conditions as the LacI-enhanced strain of *Synechocystis* but without LacI, we constructed an engineered strain with the chloramphenicol resistance-only inserted into the neutral site. This chloramphenicol resistance-only strain of *Synechocystis* was constructed and confirmed in the same way as the LacI-enhanced strain.

### Construction and characterization of the P*trc-*derived promoter library in *E. coli*

**Figure 3:**
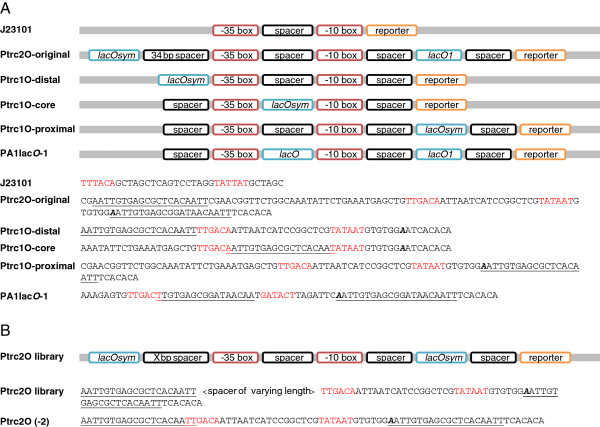
**P*trc*-library promoter design overview and sequences. A**. Control promoters. J23101 corresponds to BioBrick BBa_J23101 (iGEM Registry [34]) and is a constitutive control promoter without *lac* operator (*lacO*). P*trc*2O-original is the P*trc*2O version previously characterized [25] with *lacO1* in a proximal position and the symmetric *lacO* (*lacO*sym) in a distal position. P*trc*1O-distal, -core, and-proximal are P*trc*-derived promoters with only one *lacO*sym in the indicated positions. PA1*lacO*-1 is a previously used promoter with *lacO* in the core and proximal positions [36]. **B**. Overview of the P*trc*2O-library. The spacer sequence between the −35 box and the distal *lacO*sym varies in length from −2 bp (where 2 bp of *lacO*sym is part of the −35 box, as illustrated) to 21 bp. The variable-length spacer sequence is derived from the P*trc*2O-original spacer sequence. Transcriptional start sites are marked in bold italic.

To investigate the limited repression of P*trc*, the limited induction of P*trc*2O that we previously encountered in *Synechocystis*[25], and the characteristics of LacI-mediated DNA-looping in cyanobacteria, we designed and constructed a series of P*trc*-derived promoters. This collection consists of control promoters (Figure 3A) and the P*trc*2O-library itself (Figure 3B). Among the control promoters BBa_J23101 serves as a constitutive control and reference. PA1*lacO*-1 [36] is a strongly-repressed yet highly inducible promoter in *E. coli* that was recently found to be both repressed and inducible in *Synechocystis*[28]. The original P*trc*2O promoter works as a reference to our previous experiment [25] and P*trc*1O-distal as a P*trc*-derived promoter with the symmetric *lac* operator *(lacO*sym) inserted immediately upstream of the −35 element. P*trc*1O-core with a slightly truncated *lacO*sym inserted in the core of the promoter serves as a control for PA1*lacO*-1 that also contains a core-located *lacO*. P*trc*1O-proximal is identical to the original P*trc*[24] that we previously characterized [25] except for that we switched the proximal *lacO1* with *lacO*sym. If the low repressibility of P*trc* that we previously observed in *Synechocystis* is due to lack of LacI binding *lacO1*, we should observe an increase in repression by switching *lacO1* for the about 6-fold stronger *lacO*sym operator [13]. P*trc*1O-proximal also serves as a proximal-*lacO*sym-only control for the P*trc*2O-library. The P*trc*2O-library was built based on P*trc*2O-original but, identically to P*trc*1O-proximal, the proximal *lacO*1 has been exchanged with *lacO*sym to enhance repression, and the distance between the primary proximal and the secondary distal *lacO*sym was varied by varying the length of a spacer sequence between the −35 element and the distal *lacO*sym. This spacer sequence varies in length from −2 bp, where the distal *lacO*sym goes 2 bp into the −35 element (Figure 3B), and 21 bp, and consists of the sequence previously used in the 34 bp spacer of P*trc*2O-original. For characterization, the P*trc*-derived promoter library was cloned with the same EYFP reporter construct as the constitutive promoters.

**Figure 4:**
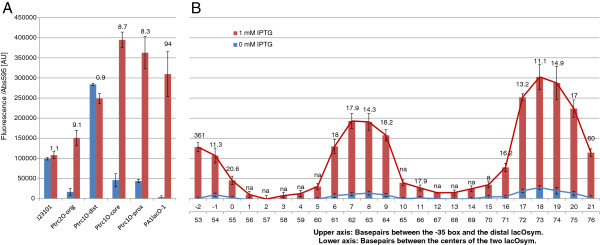
**Promoter activities per cell under non-induced (0 mM IPTG) and induced (1 mM IPTG) conditions, characterized using an EYFP reporter construct carried on pPMQAK1 **[25]**in *Escherichia coli *NEB5α F’ *I***^***q ***^**(New England Biolabs). A**. Control promoters. J23101 corresponds to BioBrick BBa_J23101 (iGEM Registry [34]) and is a constitutive control promoter without *lac* operator (*lacO*). P*trc*2O-original is the P*trc*2O version previously characterized [25] with *lacO1* in the proximal position and the symmetric *lacO* (*lacO*sym) in the distal position. P*trc*1O-distal, -core, and-proximal are P*trc*-derived promoters with only one *lacO*sym at the indicated position. PA1*lacO*-1 is a previously used promoter with *lacO* in the core and proximal positions [36]. **B**. The P*trc*2O-library. The length of the spacer sequence between the −35 box and the distal *lacO*sym is indicated on the upper x-axis and the distance between the two *lacO*sym is indicated on the lower x-axis. Promoter activities per cell are given in fluorescence/absorbance at 595 nm in arbitrary units [AU]. Error bars indicate standard error of the mean (n = 6). The numbers on top of the columns are the induction ratios. na, not available (repressed fluorescence under detection limit). IPTG, Isopropyl β-D-1-thiogalactopyranoside.

The P*trc*-derived promoter library was characterized in the *E. coli* NEB5α F’ *lacI*^q^ strain (New England Biolabs) using a setup with LB medium in 96-well plates supplemented with relevant antibiotics and 0 mM or 1 mM IPTG, for non-induced and induced conditions, respectively. For the control promoters (Figure 4A), we could observe that the constitutive control BBa_J23101 displays stable expression under both induced conditions and non-induced. P*trc*2O-orig and P*trc*1O-prox display the same limited repression as previously characterized [25] where P*trc*2O-orig is more strongly repressed than P*trc*1O-prox. The distal *lacO*sym control promoter P*trc*1O-dist displays no detectable repression whereas P*trc*1O-core displays repression similar to P*trc*1O-prox. PA1*lacO*-1, which contains a core and a proximal *lacO*, displays strong repression yet highly inducible expression as expected from its original characterization [36]. For the P*trc*2O-library (Figure 4B), repression is a periodic function of the distance between the two *lacO*sym with a period of 11 bp, approximately the expected bp per turn of the chromatin DNA in vivo [20], for both the non-induced and the induced conditions. Maximal repression is represented by the two troughs of the pattern, separated by one turn of DNA or 11 bp, and minimal repression is represented by the two peaks of the pattern, also separated by one turn of DNA. Maximal repression is not observed for inter-*lacO*sym distances that are even multiples of 11 bp, corresponding to 55 and 66 bp, which is expected to be optimal if the average number per turn over the promoter sequence was 11 bp. Instead, repression maxima appear at 57 and 67 or 68 bp, a deviation of 2 bp for the shorter loop and 1-2 bp for the longer one. Another observed trend for both conditions is that repression decreases with the distance of the secondary distal *lacO*sym from the promoter and the primary proximal *lacO*sym. The induction ratio values (Figure 4A and B) should be interpreted with caution for highly repressed promoters, as these values are close to the detection limit and even small variation due to noise will give large effects on the ratio.

### Characterization of the P*trc-*derived promoter library in the LacI-expressing strain of *Synechocystis*

**Figure 5:**
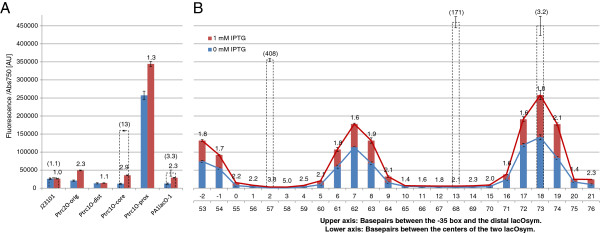
**Promoter activities per cell under non-induced (0 mM IPTG) and induced (1 mM IPTG) conditions, characterized using an EYFP reporter construct carried on pPMQAK1 **[25]**in an engineered *Synechocystis *PCC 6803 strain expressing LacI (this work). A**. Control promoters. J23101 corresponds to BioBrick BBa_J23101 (iGEM Registry [34]) and is a constitutive control promoter without *lac* operator (*lacO*). P*trc*2O-original is the P*trc*2O version previously characterized [25] with *lacO1* in the proximal position and the symmetric *lacO* (*lacO*sym) in the distal position. P*trc*1O-distal,-core, and-proximal are P*trc*-derived promoters with only one *lacO*sym at the indicated position. PA1*lacO*-1 is a previously used promoter with *lacO* in the core and proximal positions [36]. **B**. The P*trc*2O-library. The length of the spacer sequence between the−35 box and the distal *lacO*sym is indicated on the upper x-axis and the distance between the two *lacO*sym is indicated on the lower x-axis. Promoter activities per cell are given in fluorescence/absorbance at 750 nm in arbitrary units [AU]. Broken line columns represent promoter activity per cell for the corresponding promoter tested in the chloramphenicol resistance-only strain of *Synechocystis* (LacI negative, this work). Error bars indicate standard error of the mean for the *Synechocystis* PCC 6803 strain expressing LacI (n = 4) and the chloramphenicol resistance-only *Synechocystis* strain (n = 2). The numbers on top of the normal columns are the induction ratios, the numbers in parenthesis on top of the broken columns are the repression ratios. IPTG, Isopropyl β-D-1-thiogalactopyranoside.

The complete P*trc-*derived promoter library (Figure 3A and B) was characterized in the LacI-expressing strain of *Synechocystis* using a setup with BG11 medium in 96-well plates supplemented with relevant antibiotics and 0 mM or 1 mM IPTG, for non-induced and induced conditions, respectively. As a control for promoter activity in the absence of LacI, selected promoters (BBa_J23101, P*trc*1O-core, PA1*lacO*-1 and the P*trc*2O-library constructs P*trc*2O-2, 13 and 18) were characterized also in the Chloramphenicol resistance-only strain of *Synechocystis*. This was done similarly as the characterization in the LacI-expressing strain of *Synechocystis*, but a setup with BG11 in 13 mL growth tubes was used instead of 96-well plates. For the control promoters (Figure 5A) we could observe that the constitutive control BBa_J23101 displays stable expression under both non-induced and induced conditions. Further, when comparing BBa_J23101-driven expression between the constitutive promoter assay (Figure 1) and the LacI-repression assay (Figure 5A), we observe that the level of fluorescence per cell is very similar even though the conditions were different. Wild-type *Synechocystis* was used in the first case whereas the LacI-enhanced and the Chloramphenicol resistance-only strains were used in the second, the starter seed cultures were treated differently in the two cases, and 5 ml 6-well plate cultures were used in the first assay whereas 200 μl 96-well plate cultures or 1 mL cultures in 13 mL growth tubes were used in the second. Hence, under the conditions tested here, BBa_J23101 is stably constitutive in *Synechocystis*. For P*trc*2O-orig we largely reproduced our previous characterization result [25], even though the assays differed in the expression of LacI and the growth conditions. There is a small difference in that P*trc*2O-orig is not as well-repressed here, which could indicate that the levels of LacI are somewhat lower than in our previous study. The P*trc*1O-prox, which differs from the P*trc* we used previously [25] in that *lac*O1 of P*trc* has been switched for the more efficient *lac*Osym, largely behaves like P*trc* under non-induced and induced conditions, being very poorly repressed. This contrasts with the situation in *E. coli* where it is more efficiently repressed. The distal *lacO*sym promoter P*trc*1O-dist does not show strong activity, whereas it does in *E. coli*, and displays no difference between non-induced or induced conditions, there agreeing with the *E. coli* results. The core *lacO*sym promoter P*trc*1O-core behaves very similarly to PA1*lacO*-1, which carries one slightly truncated *lacO* in the core and *lacO1* in the proximal position, both displaying limited repression and induction. This contrasts strongly with the situation in *E. coli* where PA1*lacO*-1 is highly repressed yet displays strong expression under induction. When tested without LacI in the Chloramphenicol resistance-only strain of *Synechocystis*, P*trc*1O-core displays 13-times higher activity than under LacI-repressed conditions. This shows that the lack of induction in *Synechocystis* is not due to an inherently weak P*trc*1O-core promoter but due to the presence of induced LacI. Still, the activity of P*trc*1O-core is weaker than that of P*trc*1O-prox, even when comparing P*trc*1O-core without LacI and P*trc*1O-prox with non-induced LacI (Figure 5B). However in *E. coli*, P*trc*1O-core and P*trc*1O-prox are very similar under both repressed and induced conditions (Figure 4A). PA1*lacO*-1 however, displays a relatively low activity in *Synechocystis* even when LacI is absent (Figure 5A), and hence cannot be expected to be induced to higher levels. For the P*trc*2O-library (Figure 5B), the repression pattern is similar to the situation in *E. coli*, displaying periodic repression as a function of the distance between the two *lacO*sym with a period of 11 bp. Also as in *E. coli*, maximal repression can be observed for the troughs in the periodic repression pattern, minimal repression is represented as the two expression peaks separated by 11 bp at the same positions as in *E. coli*, and repression efficiency decreases with the distance of the secondary distal *lacO*sym to the promoter and the primary proximal operator. However, even though repression of the P*trc*2O-library is very effective under both non-induced and induced conditions for optimal *lacO*sym positions, it is very ineffective in the least optimal positions represented by the expression peaks, even under non-induced conditions. This differs strongly from the more efficiently repressed non-optimal distal *lacO*sym positions in *E. coli*, which, even though they are clearly visible as expression peaks, are repressed even under non-induced conditions. Further, repression in the geometrically-favored configurations is represented in *Synechocystis* by much wider troughs than in *E. coli*, for both conditions. This contrasts with the most unfavorable looping-geometries, represented by the narrower expression peaks in *Synechocystis* than in *E. coli*. To test if the very efficient repression for favorable DNA-looping geometries in *Synechocystis* is due to non LacI-related promoter inhibition of the P*trc*2O-library, we characterized the activity of P*trc*2O-2 and 13 in the absence of LacI in the Chloramphenicol resistance-only strain. These two highly repressed promoters, which are located in the centers of the repression troughs, display very high promoter activity in the absence of LacI (Figure 5B). Hence, they work as very high dynamic range LacI-regulated promoters, if expression could only be efficiently induced. Conversely, to test if the very non-efficient repression for non-favorable DNA-looping geometries in *Synechocystis* is due to non LacI-related promoter activation effects on the P*trc*2O-library, we also characterized the activity of P*trc*2O-18 in the absence of LacI. This inefficiently repressed promoter displayed a close to identical activity to the P*trc*2O-13 promoter, illustrating that the lack of repression of the P*trc*2O-library for non-favorable DNA-looping geometries in *Synechocystis* is not due to inherently stronger promoters. Taken together, these results show that the periodical repression pattern of the P*trc*2O-library in the LacI-expressing strain of *Synechocystis* is due to LacI-facilitated DNA-looping.

### Characterization of the proximal *lacO*-library in *E. coli* DH5αZ1 and *Synechocystis*

Differences in how the *E. coli* and the *Synechocystis* RNAP interact with LacI bound to the proximal region of *trc*-derived promoters could be investigated by comparing IPTG-induction or LacI-repression patterns of a proximal *lacO*-library between *E. coli* and *Synechocystis*. Because induction or repression ratios of the same promoters are studied, expression differences between promoters caused by differences in the 5′-UTRs will be cancelled, and the ratios will reflect the efficiencies of LacI repression for specific promoters. Such induction or repression ratios of promoter libraries where the position of single *lac* operators relative to the promoter was varied by single base-pairs have been studied before to investigate variation in LacI repression [19,20]. We reason that a potential difference in the DNA-binding activity between the *E. coli* and the *Synechocystis* RNAP might be visible in differences in the pattern of this ratio. Therefore, we designed a promoter library in which we vary the distance between the center of a symmetric *lacO* and the TSS of a P*trc* core promoter from −2 to +18 bp by adjusting the length of a spacer in 1 bp steps and from +18 to +24 bp in 2 bp steps (Table 1). We inserted this proximal *lacO*-library into an *E. coli* strain that expresses high amounts of LacI repressor, DH5αZ1, to enable the calculation of IPTG-induction ratios, and into both the LacI-expressing *Synechocystis* strain and the chloramphenicol resistance-only strain, to enable the calculation of both IPTG-induction and LacI-repression ratios.

**Table 1:** Promoter DNA sequences

**Promoter name**	**Sequence 5′ to 3′**
BBa_J23100	TTGACGGCTAGCTCAGTCCTAGGTACAGTGCTAGC
BBa_J23101	TTTACAGCTAGCTCAGTCCTAGGTATTATGCTAGC
BBa_J23102	TTGACAGCTAGCTCAGTCCTAGGTACTGTGCTAGC
BBa_J23105	TTTACGGCTAGCTCAGTCCTAGGTACTATGCTAGC
BBa_J23106	TTTACGGCTAGCTCAGTCCTAGGTATAGTGCTAGC
BBa_J23109	TTTACAGCTAGCTCAGTCCTAGGGACTGTGCTAGC
BBa_J23113	CTGATGGCTAGCTCAGTCCTAGGGATTATGCTAGC
BBa_J23114	TTTATGGCTAGCTCAGTCCTAGGTACAATGCTAGC
P*nirA*	CAAGCTCAGAATGCTGCGGGGAGAAGGGCAACTCCCCACCAGCCCCAAATTTTTGCTGGCGATAAATATTTTTCGGTTTAATTGTTCACAAAGCTTTTTGAATTTGAGTTTATAGAAATTTATTGGCTGGTAATGCTTTTTTGCCCCCCTGCTGGACTTCATTGATCCTTGCCTATACCATCAATATCATTGGTCAATAATGATGATGATTGACTAAAACATGTTTAACAAAATTTAACGCATATGCTAAATGCGTAAACTGCATATGCCTTGGCTGAGTGTAATTTACGTTACAAATTTTAACGAAACGGGAACCCTATATTGATCTCTACTGTTATCTGGCT
P*petE*	TCATAGCGGTTGCCCAATCTAACTCAGGGAGCGACTTCAGCCCACAAAAAACACCACTGGGCCTACTGGGCTATTCCCATTATCATCTACATTGAAGGGATAGCAAGCTAATTTTTATGACGGCGATCGCCAAAAACAAAGAAAATTCAGCAATTACCGTGGGTAGCAAAAAATCCCCATCTAAAGTTCAGTAAATATAGCTAGAACAACCAAGCATTTTCGGCAAAGTACTATTCAGATAGAACGAGAAATGAGCTTGTTCTATCCGCCCGGGGCTGAGGCTGTATAATCTACGACGGGCTGTCAAACATTGTGATACCATGGGCAGAAGAAAGGAAAAACGTCCCTGATCGCCTTTTTGGGCACGGAGTAGGGCGTTACCCCGGCCCGTTCAACCACAAGTCCCTATAGATACAATCGCCAAGAAGT
P*rnpB*	TTCAATGCGGTCCAATACCTCCCCTGCCCAACTGGGTAAGCTCGCGGCTCCACTGAGTAATACAGACAAGGCTAAACAGGCAAATTTTTTCATTGGTCAACTCCTAGCACCAATTTCCCAAGACTACGGAGGGGGCAATGAAGTTTCAATTAATTGGGGTCACAAACCACAGCGGCCTATGGCTCTAATCAATGGCACACTAGAAAAA
P*trc*2O-(−2)	AATTGTGAGCGCTCACAATTGACAATTAATCATCCGGCTCGTATAATGTGTGGAATTGTGAGCGCTCACAATTTCACACA
P*trc*2O-(−1)	AATTGTGAGCGCTCACAATTTGACAATTAATCATCCGGCTCGTATAATGTGTGGAATTGTGAGCGCTCACAATTTCACACA
P*trc*2O-0	AATTGTGAGCGCTCACAATTTTGACAATTAATCATCCGGCTCGTATAATGTGTGGAATTGTGAGCGCTCACAATTTCACACA
P*trc*2O-1	AATTGTGAGCGCTCACAATTGTTGACAATTAATCATCCGGCTCGTATAATGTGTGGAATTGTGAGCGCTCACAATTTCACACA
P*trc*2O-2	AATTGTGAGCGCTCACAATTTGTTGACAATTAATCATCCGGCTCGTATAATGTGTGGAATTGTGAGCGCTCACAATTTCACACA
P*trc*2O-3	AATTGTGAGCGCTCACAATTCTGTTGACAATTAATCATCCGGCTCGTATAATGTGTGGAATTGTGAGCGCTCACAATTTCACACA
P*trc*2O-4	AATTGTGAGCGCTCACAATTGCTGTTGACAATTAATCATCCGGCTCGTATAATGTGTGGAATTGTGAGCGCTCACAATTTCACACA
P*trc*2O-5	AATTGTGAGCGCTCACAATTAGCTGTTGACAATTAATCATCCGGCTCGTATAATGTGTGGAATTGTGAGCGCTCACAATTTCACACA
P*trc*2O-6	AATTGTGAGCGCTCACAATTGAGCTGTTGACAATTAATCATCCGGCTCGTATAATGTGTGGAATTGTGAGCGCTCACAATTTCACACA
P*trc*2O-7	AATTGTGAGCGCTCACAATTTGAGCTGTTGACAATTAATCATCCGGCTCGTATAATGTGTGGAATTGTGAGCGCTCACAATTTCACACA
P*trc*2O-8	AATTGTGAGCGCTCACAATTATGAGCTGTTGACAATTAATCATCCGGCTCGTATAATGTGTGGAATTGTGAGCGCTCACAATTTCACACA
P*trc*2O-9	AATTGTGAGCGCTCACAATTAATGAGCTGTTGACAATTAATCATCCGGCTCGTATAATGTGTGGAATTGTGAGCGCTCACAATTTCACACA
P*trc*2O-10	AATTGTGAGCGCTCACAATTAAATGAGCTGTTGACAATTAATCATCCGGCTCGTATAATGTGTGGAATTGTGAGCGCTCACAATTTCACACA
P*trc*2O-11	AATTGTGAGCGCTCACAATTGAAATGAGCTGTTGACAATTAATCATCCGGCTCGTATAATGTGTGGAATTGTGAGCGCTCACAATTTCACACA
P*trc*2O-12	AATTGTGAGCGCTCACAATTTGAAATGAGCTGTTGACAATTAATCATCCGGCTCGTATAATGTGTGGAATTGTGAGCGCTCACAATTTCACACA
P*trc*2O-13	AATTGTGAGCGCTCACAATTCTGAAATGAGCTGTTGACAATTAATCATCCGGCTCGTATAATGTGTGGAATTGTGAGCGCTCACAATTTCACACA
P*trc*2O-14	AATTGTGAGCGCTCACAATTTCTGAAATGAGCTGTTGACAATTAATCATCCGGCTCGTATAATGTGTGGAATTGTGAGCGCTCACAATTTCACACA
P*trc*2O-15	AATTGTGAGCGCTCACAATTTTCTGAAATGAGCTGTTGACAATTAATCATCCGGCTCGTATAATGTGTGGAATTGTGAGCGCTCACAATTTCACACA
P*trc*2O-16	AATTGTGAGCGCTCACAATTATTCTGAAATGAGCTGTTGACAATTAATCATCCGGCTCGTATAATGTGTGGAATTGTGAGCGCTCACAATTTCACACA
P*trc*2O-17	AATTGTGAGCGCTCACAATTTATTCTGAAATGAGCTGTTGACAATTAATCATCCGGCTCGTATAATGTGTGGAATTGTGAGCGCTCACAATTTCACACA
P*trc*2O-18	AATTGTGAGCGCTCACAATTATATTCTGAAATGAGCTGTTGACAATTAATCATCCGGCTCGTATAATGTGTGGAATTGTGAGCGCTCACAATTTCACACA
P*trc*2O-19	AATTGTGAGCGCTCACAATTAATATTCTGAAATGAGCTGTTGACAATTAATCATCCGGCTCGTATAATGTGTGGAATTGTGAGCGCTCACAATTTCACACA
P*trc*2O-20	AATTGTGAGCGCTCACAATTAAATATTCTGAAATGAGCTGTTGACAATTAATCATCCGGCTCGTATAATGTGTGGAATTGTGAGCGCTCACAATTTCACACA
P*trc*2O-21	AATTGTGAGCGCTCACAATTCAAATATTCTGAAATGAGCTGTTGACAATTAATCATCCGGCTCGTATAATGTGTGGAATTGTGAGCGCTCACAATTTCACACA
P*trc*2O-orig	CGAATTGTGAGCGCTCACAATTCGAACGGTTCTGGCAAATATTCTGAAATGAGCTGTTGACAATTAATCATCCGGCTCGTATAATGTGTGGAATTGTGAGCGGATAACAATTTCACACA
P*trc*1O-dist	AATTGTGAGCGCTCACAATTTTGACAATTAATCATCCGGCTCGTATAATGTGTGGAATCACACA
P*trc*1O-core	AAATATTCTGAAATGAGCTGTTGACAATTGTGAGCGCTCACAATATAATGTGTGGAATCACACA
P*trc*1O-prox	CGAACGGTTCTGGCAAATATTCTGAAATGAGCTGTTGACAATTAATCATCCGGCTCGTATAATGTGTGGAATTGTGAGCGCTCACAATTTCACACA
PA1*lacO*-1	AAAGAGTGTTGACTTGTGAGCGGATAACAATGATACTTAGATTCAATTGTGAGCGGATAACAATTTCACACA
PtrcOs-prox-8	TTGACAATTAATCATCCGGCTCGTATAATGTGTAATTGTGAGCGCTCACAATT
PtrcOs-prox-9	TTGACAATTAATCATCCGGCTCGTATAATGTGTGAATTGTGAGCGCTCACAATT
PtrcOs-prox-10	TTGACAATTAATCATCCGGCTCGTATAATGTGTGGAATTGTGAGCGCTCACAATT
PtrcOs-prox-11	TTGACAATTAATCATCCGGCTCGTATAATGTGTGGAAATTGTGAGCGCTCACAATT
PtrcOs-prox-12	TTGACAATTAATCATCCGGCTCGTATAATGTGTGGAGAATTGTGAGCGCTCACAATT
PtrcOs-prox-13	TTGACAATTAATCATCCGGCTCGTATAATGTGTGGAGTAATTGTGAGCGCTCACAATT
PtrcOs-prox-14	TTGACAATTAATCATCCGGCTCGTATAATGTGTGGAGTCAATTGTGAGCGCTCACAATT
PtrcOs-prox-15	TTGACAATTAATCATCCGGCTCGTATAATGTGTGGAGTCGAATTGTGAGCGCTCACAATT
PtrcOs-prox-16	TTGACAATTAATCATCCGGCTCGTATAATGTGTGGAGTCGTAATTGTGAGCGCTCACAATT
PtrcOs-prox-17	TTGACAATTAATCATCCGGCTCGTATAATGTGTGGAGTCGTCAATTGTGAGCGCTCACAATT
PtrcOs-prox-18	TTGACAATTAATCATCCGGCTCGTATAATGTGTGGAGTCGTCCAATTGTGAGCGCTCACAATT
PtrcOs-prox-20	TTGACAATTAATCATCCGGCTCGTATAATGTGTGGAGTCGTCCAGAATTGTGAGCGCTCACAATT
PtrcOs-prox-22	TTGACAATTAATCATCCGGCTCGTATAATGTGTGGAGTCGTCCAGACAATTGTGAGCGCTCACAATT
PtrcOs-prox-24	TTGACAATTAATCATCCGGCTCGTATAATGTGTGGAGTCGTCCAGACTCAATTGTGAGCGCTCACAATT

**Figure 6:**
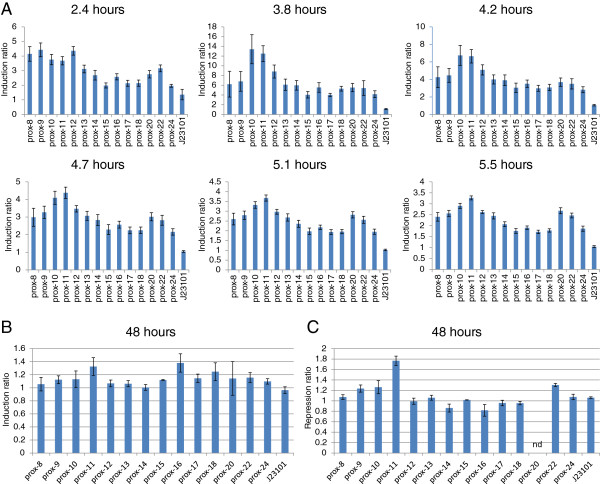
**Patterns of induction and repression ratios for the proximal *lacO *promoter library characterized using a SGFP2 reporter construct carried on pPMQAK1 **[25]**.** The number in prox-# indicates the distance between the center of the proximal symmetric *lac* operator and the transcriptional start site of a P*trc* core promoter. The time in hours represents time from start of experiment. J23101 corresponds to BioBrick BBa_J23101 (iGEM Registry [34]) and is a constitutive control promoter without *lac* operator. **A**. Time-course study of induction ratios (fluorescence per cell values of 2 mM IPTG induced cultures/the values of the non-induced cultures) in *E. coli* DH5αZ1. Error bars represent the estimation of the standard error of the mean for the induction ratios (n = 6). **B**. Induction ratios in the *Synechocystis* PCC 6803 strain expressing LacI (this work). Error bars represent the estimation of the standard error of the mean for the induction ratios (n = 2). **C**. Repression ratios (fluorescence per cell values of cultures from the chloramphenicol resistance-only *Synechocystis* strain (LacI negative, this work)/the values of cultures from the *Synechocystis* PCC 6803 strain expressing LacI). Error bars represent the estimation of the standard error of the mean for the repression ratios (n = 2). nd, not determined.

The characterization of the proximal *lacO*-library in *E. coli* DH5αZ1 was performed similarly to the characterization of the P*trc-*derived promoter library in NEB5α, but a key difference is that the proximal *lacO***-**library was followed in time by measuring the same plates at different time-points and then continuing the experiment. The induction ratios were calculated from the fluorescence/absorbance values of the induced/non-induced cultures and the standard errors of the ratios were estimated (Figure 6A). An apparent steady-state of fluorescence/cell for most of the promoters (data not shown) was observed at ca 3.8 hours after the start of the experiment. At this time point, the induction ratios are also the highest; the J23101 constitutive control has reached its expected ratio of ca 1 and the ratios diminish progressively in value at later time-points. The induction ratio pattern established from ca 3.8 hours remains, however, also at later time points (Figure 6A). As shown in previous studies of induction and repression ratios of libraries of proximal *lacO*-promoters in *E. coli*[19], the *lacO* centered at +11 relative to the TSS (prox-11 in Figure 6A) provides the most efficient repression, closely followed by the +10 promoter. The +11 position corresponds to the position of *lacO1* in the wild-type *E. coli lac* promoter [10] and is one helix turn away from the TSS, putting LacI on the same side of the DNA as the RNAP.

The characterization of the proximal *lacO***-**library in *Synechocystis* was performed similarly to the characterization of the P*trc-*derived promoter library, but 13 mL growth tubes were used instead of 96-well plates, and, as compared with the *E. coli* DH5αZ1 experiment, only one time-point was used. The induction ratios were calculated from the fluorescence/absorbance values of the induced/non-induced LacI-expressing *Synechocystis* cultures and the standard errors of the ratios were estimated (Figure 6B). The repression ratios were calculated from the fluorescence/absorbance values of the chloramphenicol resistance-only cultures/non-induced LacI-expressing *Synechocystis* cultures and the standard errors of the ratios were estimated (Figure 6C). First, we observe that the ratios are much smaller, with a smaller difference between the different promoters, than in *E. coli* DH5αZ1. However, both the induction and the repression ratios in *Synechocystis* are similar to the induction ratios in *E. coli* DH5αZ1. Also in *Synechocystis*, the centered +11 position of the *lacO* relative to the TSS produces the most efficiently repressed proximal *lacO* promoter. To summarize, no difference in the induction or repression patterns between *E. coli* and *Synechocystis* can be detected.

## Discussion

To elucidate the design principles of LacI-repressed promoters and DNA-looping in cyanobacteria, we constructed a P*trc*-derived promoter library consisting of single *lac* operator control promoters (Figure 3A) and dual *lacO* promoters with LacI-facilitated DNA-loop capability (Figure 3B). For characterizing the repression and induction of this library, we first characterized several artificial minimal promoters in *Synechocystis* and used this knowledge to engineer a constitutive LacI-expressing strain of *Synechocystis*. Finally, to investigate if differences between the *E. coli* and *Synechocystis* RNA polymerases would affect LacI-repression of promoters with proximally located *lacO*, we engineered and characterized a library of proximal *lacO* promoters in both *E. coli* and *Synechocystis*.

### Minimal and artificial promoters as orthogonal alternatives to native cyanobacterial promoters

As orthogonal parts can help minimize or eliminate cross-talk and unwanted interactions between parts or between parts and the chassis [7], we first set out to characterize several minimal and artificial promoters that could potentially drive the constitutive expression of LacI in *Synechocystis*. The artificial, minimal and constitutive promoters BBa_J23100, BBa_J23101, BBa_J23102, BBa_J23105, BBa_J23106, BBa_J23109, BBa_J23113 and BBa_J23114 were found to span a greater range of expression levels, high and low, as compared to the three commonly used native promoters P*nirA,* P*petE* and P*rnpB* from *Synechocystis*. The artificial promoters are derived from a consensus *E. coli* promoter, which −35 and −10 elements are almost identical to the consensus −35 and −10 boxes of *Synechocystis*[37]. These types of promoters, belonging to the group 1 promoters in cyanobacteria, are served by the primary cyanobacterial sigma factor SigA during normal growth conditions [26]. Hence, all the artificial promoters tested here can be classified as cyanobacterial group 1 promoters, and as orthogonal and minimal promoters, minimizing the risk for cross-talk with native transcription factors or sRNAs, they are expected to remain constitutive under normal growth conditions. In favor of this, the BBa_J23101 promoter was found to display stable levels of expression of EYFP per cell under the different conditions of light-intensity, culture volumes and mixing, presence of the exogenous chemical IPTG, and three chassis (wild-type, LacI-enhanced and chloramphenicol resistance-only strains of *Synechocystis*) that were used in this study. Further, the weak BBa_J23114 promoter was used with RBS* to express relatively low levels of LacI/cell (about 190 tetramers of LacI/cell) in the LacI-enhanced strain of *Synechocystis*. Therefore, we suggest that promoters from the iGEM Registry’s Anderson collection could be used as minimal orthogonal alternatives to native promoters for constitutive expression in cyanobacteria, or as core promoters for the engineering of regulated promoters. Further, as the BBa_J23101 promoter has been used as a standard reference promoter in *E. coli*[38], we propose that it could fulfill the same role in cyanobacteria. As all the P*trc*-derived promoters used in this study are close-to-consensus group 1 cyanobacterial promoters, orthogonal to *Synechocystis,* and minimal in the sense that they only contain the necessary elements for recognition by RNAP and regulation by LacI, they are also expected to be constitutive in the absence of LacI, which we showed herein for selected members of the library. The orthogonality also means that they should display no or minimal cross-talk with native transcription factors and sRNAs. However, to truly test the variation of expression, and find constitutive promoters with minimal variation of expression, a more rigorous study of diverse environmental conditions than the present work is warranted. For example, cyanobacteria naturally grow in day and night cycles and have a circadian rhythm that affects a large part of the transcriptome, seemingly mainly through modulating DNA topology [39]. How or if circadian rhythms would modulate orthogonal promoters in cyanobacteria would have to be tested. Further, a change in DNA topology could change the relative spatial positioning or phasing on the helix of a promoter and the binding site of a transcription factor, such as LacI, possibly rendering repression even by an orthogonal repressor circadian rhythm-dependent.

Other environmental conditions are also relevant to test for orthogonal promoters, such as higher variations in light intensity and duration of darkness, changes in salinity and temperature. It is well known that different environmental variations give rise to sigma-factor switching, a phenomenon that helps cyanobacteria adapt to diverse changes by switching between different sigma-factors that activate the expression of stress responses [26]. To further decouple synthetic circuits from cell regulation and phenomena such as sigma-switching, system orthogonality could be increased by using orthogonal RNAP and their corresponding promoters, which are decoupled from the native transcriptional machinery of the host. For example, the bacteriophage T7 RNAP can be engineered to produce orthogonal genetic circuits incorporating several mutually orthogonal T7 RNAP [40] or orthogonal split T7 RNAP [41].

### LacI-repressed promoters in *E. coli* and *Synechocystis*

Since the following analysis builds on comparisons of promoter repression patterns, it is important to note that expression also depends on the 5′ untranslated region, the coding sequence of the particular gene of interest (GOI) and their combined interactions. The folding status of the 5′ UTR and in particular the ribosome binding site (RBS) strongly affects translation initiation [42]. Since the first part of the GOI sequence is located close to the 5′ UTR on the mRNA, its identity will affect potential mRNA secondary structures involving the RBS. Further, internal GOI sequence elements such as codon identities and secondary structures will affect translation elongation [43]. Consequently, when comparing promoter strengths indirectly through the expression of a reporter gene it only makes sense to compare constructs with the same 5′ UTR and coding sequence. In this study, the same reporter cassette featuring the same RBS and coding sequence is used for all promoters within a library. However, some of the promoters in the P*trc*-derived library differ in the sequences that come after the transcription start site (TSS), meaning that these promoters will have slightly different 5′ UTRs (Figures 3A and B). Among these promoters, P*trc*1O-proximal and the whole P*trc*2O-library, except P*trc*2O-original, share the same 5′ UTR. Further, P*trc*1O-distal and P*trc*1O-core share the same 5′ UTR whereas BBa_J23101, P*trc*2O-original and PA1*lacO*-1 do not share 5′ UTRs. However, the induction ratios can still be compared between promoters that do not share exact 5′ UTRs as 5′ UTR effects that affect both the induced state and the non-induced state will be cancelled. This is true also for the proximal *lacO* promoter library (Table 1), where all promoters differ slightly in their 5′ UTRs but only induction or repression ratios are compared. Finally, it should be noted that the fluorescence from promoters that are highly repressed are close to the detection limit, meaning that even small amounts of variation due to noise will affect the induction or repression ratio values strongly. Hence, in general caution should be exercised when comparing promoter activity ratios for strongly repressed promoters.

We engineered a strain of *Synechocystis* to express similar levels of LacI as in our previous study [25] for repression of the P*trc*-derived promoter library. Also, we previously observed that P*trc* and P*trc*2O behave as constitutive promoters of approximately the same strength in the absence of heterologously expressed LacI, displaying stable expression levels irrespective of the presence of IPTG. Therefore, and since we are herein interested in analyzing the patterns of repression and induction depending on *lacO* location(s), we did not analyze the majority of the P*trc*-derived promoter library in the absence of LacI. Further, by comparing repression and induction levels of P*trc*2O (here termed P*trc*2O-original to distinguish it from the other P*trc*2O promoters) in *Synechocystis* between the present (Figure 5A) and our previous study [25], we observe a similar pattern but that the induction/repression ratio is about two-fold lower in this study. As the two studies differ in both the characterization growth conditions and the LacI expression cassettes we cannot confidently conclude the reason for this difference, but the discrepancy leads us to estimate that the levels of LacI in the present study are similar to or lower than in our previous study. We estimated the number of LacI tetramers per cell to be about 190 for the engineered LacI-expressing strain developed here, so the number can be expected to be higher than 190 in our previous study [25].

To investigate if a lack of LacI binding the proximal *lacO1* of P*trc* was the reason for the limited repression in *Synechocystis* that we previously observed [25], we exchanged *lacO1* with the stronger *lacO*sym operator. However, no increase in the induction ratio was observed (Figure 5A). This however, could be due to the possibly lower levels of LacI in this study, or that the 1 bp shorter *lacO*sym places LacI in a spatially less optimal position relative to the promoter’s TSS than *lacO1*. Nonetheless, when comparing the repression and induction results of P*trc*1O-proximal between *E. coli* (Figure 4A) and *Synechocystis* (Figure 5A) we managed to confirm our previous results [25] that the proximal *lacO* position downstream to and overlapping 1 bp with the TSS leads to more efficient repression in *E. coli* as compared to in *Synechocystis*. Another study observed the same lack of repression of P*trc* in *Synechocystis* when using another construct to express LacI [28]. This reproducibility, and the fact that the levels of LacI expressed in the LacI-enhanced strain of *Synechocystis* are sufficient for much more effective repression of other *lacO* promoters (Figures 5A and B) lead us to hypothesize that the lack of repression of P*trc* and its twin P*trc*1O-proximal in *Synechocystis* is due to the characteristics of RNAP, and not due to the lack of LacI. In bacteria, the RNAP holoenzyme consists of the α_2_, β, β’ and ω subunits plus a σ-factor that gives promoter specificity [44]. A time-resolved *E. coli* RNAP-promoter footprinting study showed that RNAP makes first contact with the upstream part of the promoter, and then extends downstream towards the −10 element, the TSS and all the way to about +20 bp from the TSS as it proceeds from a closed to an open complex [45]. It was recently shown that the RNAP clamp, consisting of pincer-like parts of the β’ and the β subunits, progressively closes around the double stranded DNA downstream from the TSS as the complex approaches transcription initiation, and then remains closed during elongation [46]. In cyanobacteria, the β’ subunit has been split up in two parts: γ, which corresponds to the N-terminal part of the bacterial β’ subunit, and β’, which corresponds to the C-terminal part [31,32]. It was observed in a later study that enteric and cyanobacterial RNAP transcribe promoters differently, and differences in RNAP architecture, including a large insertion in the β’ subunit was suggested to be the cause [47]. Since then, these differences between the enteric and the cyanobacterial RNAP has been suggested to explain differences in promoter activity due to single nucleotide mutations [48] and more recently differences in elongation rate and fidelity [49].

Hence we hypothesize that the differences between the *E. coli* RNAP β’ subunit and the cyanobacterial RNAP γ + β’ subunits could explain the apparent decreased sensitivity of *Synechocystis* RNAP towards LacI binding the proximal *lac* operator of P*trc* and P*trc*1O-proximal. Since the β’ and β clamp of enteric RNAP closes around the DNA downstream of the TSS during transcription initiation, and β’ is the subunit that differs the most between cyanobacteria and *E. coli*, we speculate that the RNAP clamp of cyanobacteria may possess an increased stability or a different DNA footprint than enteric RNAP, blocking or removing a proximally bound LacI more effectively. If the DNA footprint differs, the proximal promoter binding site location for maximizing repression by a transcription factor is likely to be different for cyanobacteria as compared to enterobacteria. To test if the DNA footprint of *E. coli* and *Synechocystis* RNAP differ for the proximal promoter region close to and downstream the TSS, we designed a library of proximal *lacO* promoters in which we vary the distance between the center of a symmetric *lacO* and the TSS of a P*trc* core promoter by steps of 1 or 2 bp. We characterized the induction and/or the repression ratio patterns for this proximal promoter library in both *E. coli* and *Synechocystis*, but could not detect any differences. Hence, our results do not support the hypothesis that there is a difference in the DNA footprint between the *E. coli* and *Synechocystis* RNAP at the promoter proximal location. If the cyanobacterial RNAP possesses an increased DNA-binding stability in the promoter proximal region remains to be tested, and what the potential effect of the β’ subunit split and insertion in cyanobacteria is remains to be shown.

Finally, the fact that P*trc* can be more efficiently repressed and induced in *Synechococcus elongatus* PCC 7942 [27] than in *Synechocystis*[25,28] may simply be explained by a higher number of LacI/cell in the *Synechococcus* study than in the *Synechocystis* studies. Indeed, the numbers of LacI/cell expressed in the LacI-enhanced strain of *Synechocystis* herein presented is about five times lower than the per cell value for *E. coli* NEB5α, and about sixteen times lower than in DH5αZ1.

Recently, the in *E. coli* well-repressed yet highly inducible promoter PA1*lacO*-1 [36] was characterized in *Synechocystis* using a plasmid-carried *lacI*^*q*^ gene for expressing LacI and an ethylene-forming enzyme (EFE) as a reporter [28]. Characterization was carried out by measuring the rate of ethylene formation per volume, time and cell density and comparing IPTG-inducing and non-inducing conditions for different cell densities. The repression of PA1*lacO*-1 was found to be higher at lower cell densities and lower at higher cell densities. Further, they tested P*trc* with the same setup and found that PA1*lacO*-1 could be induced to the same level as P*trc*. To enable the comparison of a regulated high dynamic range *E. coli* promoter with its activity in *Synechocystis*, we characterized PA1*lacO*-1 with the same EYFP reporter construct as the rest of the P*trc*-derived library (Figures 3A and B). As expected, we found that PA1*lacO*-1 was strongly repressed yet highly inducible in *E. coli* (Figure 4A). However, even though we found that PA1*lacO*-1 is repressed in *Synechocystis*, in line with the previous study [28], we found that it could not be induced to the activity of P*trc*1O-prox (Figure 5A), which is almost identical in its repression and induction behavior to P*trc*[25]. Still, it is problematic to directly compare P*trc*1O-prox/P*trc* with PA1*lacO*-1 since they have slightly different 5′ UTRs, as well as comparing the two studies that use different reporters, both factors potentially affecting expression. Further, for promoter activity quantification in general, it is problematic to infer promoter activities indirectly from the expression of protein reporters. This problem can be solved by directly measuring promoter mRNA output using quantitative reverse transcription PCR. However, this poses a problem of its own as it cannot be done in living cells. Hence, it may sometimes still be preferable to characterize promoters indirectly through the use of fluorescent reporters, since this can be done in vivo. For instance, absolute quantification of promoters can be done using fluorescent protein reporters together with quantitative models to derive promoter strengths [50] or the number of RNAP that clears the promoter per second [38]. Finally, using the activity of an enzymatic protein reporter for inferring promoter activity adds an additional level of uncertainty over using fluorescent proteins, as enzymes depend on many factors, for instance substrate availability. These considerations make it difficult to speculate about the reason(s) for the difference in induced expression of P*trc*1O-prox/P*trc* versus PA1*lacO*-1 when comparing this and the previous study [28]. Further, PA1*lacO*-1 consists of a proximal *lacO1* 1 bp downstream in position as compared to P*trc* and P*trc*1O-proximal and a core *lacO* at the same position as P*trc*1O-core (Figure 3A). As the proximal *lacO* position of P*trc* and P*trc*1O-proximal has proven ineffective for repression and because PA1*lacO*-1 looks similar to P*trc*1O-core in its repression and induction pattern (Figure 5A), we agree with the previous study [28] that the core-located *lacO* appears to be causing the drop in expression. To test this, we characterized both P*trc*1O-core and PA1*lacO*-1 in the LacI-negative chloramphenicol resistance-only strain of *Synechocystis.* Indeed, both promoters were weaker than P*trc1O*-proximal, even when their activity without LacI is compared with the repressed activity of P*trc1O*-proximal (Figure 5A). Further, the activity of PA1*lacO*-1 was much lower than that of P*trc*1O-core in the absence of LacI, leading to a repression ratio of only 3.3 for PA1*lacO*-1 and 13 for P*trc*1O-core. It is possible that the slightly lower induction ratio for PA1*lacO*-1 as compared to the previous study [28] is due to lower levels of LacI/cell in this study. Finally, the reduced inherent promoter activity of P*trc*1O-core and PA1*lacO*-1 in *Synechocystis* as compared with *E. coli* further stresses our previous conclusion that tools to be used in a specific bacterium have to be functionally characterized in that particular chassis [4,25] and illustrates the need for orthogonal and hence portable RNAP systems.

### LacI-mediated DNA-looping in *E. coli* and *Synechocystis*

To investigate the effect of tetrameric LacI-cooperativity on repression and DNA-looping in cyanobacteria, we designed a P*trc*-based library with two *lacO* to enable LacI-mediated DNA looping in analogy with previous designs for *E. coli*[20,23,51]. We added an auxiliary *lacO*sym at different distances, ranging from −2 to 21 bp, upstream of the P*trc*1O-proximal −35 element, while keeping the core P*trc* promoter and proximal *lacO*sym of P*trc*1O-proximal intact. As the P*trc*2O-library and P*trc*1O-proximal share identical promoters from the −35 box to the proximal *lac* operator and further produce identical 5′ UTRs (Figures 3A and B), this enables us to directly compare them and detect effects from LacI-mediated DNA looping. Further, single *lacO*sym operators positioned upstream of a *lac*UV5 promoter −35 element have previously been shown to produce only minor effects in *E. coli* on expression in the presence of LacI and the absence of DNA-looping [51]. However, a subsequent study showed loop-independent effects of placing a single distal *lacO*sym too close to the promoter −35 element, especially for edge-to-edge distances between *lacO*sym and the −35 hexamer of 2 and 6 bp [20]. This effect was suggested to be due to interactions between LacI and the *E. coli* RNAP, or due to sequence-specific effects affecting the nearby promoter itself. Such sequence-specific effects are possible, and have been illustrated recently for cyanobacteria [5]. Therefore, the P*trc*2O-library can be compared with P*trc*1O-proximal to draw conclusions about the possible effects of DNA-looping, but caution should be taken in interpreting the data for the closest spacings of the −35 box to the distal *lacO*sym operator.

First of all, we can conclude that the repression and induction patterns of the P*trc*2O-library in *E. coli* (Figure 4B) and *Synechocystis* (Figure 5B) share many similarities. Both species produce a periodical repression and induction pattern with a period of 11 bp, illustrating that maximal repression can be achieved when both *lacO* are positioned on the same side of the DNA helix, and that minimal repression is produced when the operators are on opposite sides. To confirm that the maximal repression displayed in the troughs of the periodical pattern, and the minimal repression displayed in the peaks, are not due to promoter activity effects unrelated to LacI, we tested two highly repressed promoters and one weakly repressed promoter without LacI. Both the two highly repressed promoters were highly active in the absence of LacI, and the activity of the weakly repressed promoter was similar to the activity of the highly repressed promoters (Figure 5B), confirming that the periodical repression pattern is due to the presence of LacI. This confirms the numerous previous studies on LacI-mediated DNA looping in *E. coli*[14,16-18,20,23,51], and demonstrates that DNA-looping as a mechanism of transcriptional regulation works in cyanobacteria, displaying the same number of average bp per turn of the supercoiled DNA helix as *E. coli*. This was anticipated, as structural DNA-binding proteins like HU that have been demonstrated to facilitate LacI-mediated DNA looping in *E. coli*[52] are present also in cyanobacteria (the *Synechocystis* HU gene is *sll1712* in Cyanobase [53]). That a periodical repression pattern remains even under full IPTG induction in both *E. coli* and *Synechocystis* is not surprising. As stated previously, the binding of IPTG to LacI decreases the affinity of LacI to *lacO* approximately 1000-fold [11]. Under circumstances when LacI is in excess to its operators, or its binding to a high-affinity operator is stabilized through DNA-looping, LacI-mediated DNA-loops could remain even under induced conditions. In fact, it has been shown that saturating induction does not completely remove loops in vivo [51] or in vitro [54]. To decrease the effect of residual repression even under full IPTG-induction, concentrations of LacI should not be higher than what is required for satisfactory repression. Further, just as in *E. coli*, the repression both for the non-induced and the induced conditions decrease with longer inter-*lacO* distances in *Synechocystis*. This has been shown before in *E. coli* and illustrates the drop in contribution to the local concentration of LacI at the primary proximal repression site from the distal site for longer distances [23,55]. On the other hand, it was recently demonstrated that the LacI-mediated DNA-loop itself is enough to repress transcription from a T7 promoter positioned inside the loop [55]. As this was demonstrated using the T7 RNAP, it is not tested whether bacterial RNAP will share the same sensitivity to DNA-loops, but likely, as the polymerases have evolved to perform the same function in similar ways. Hence, the decrease in repression for longer inter-*lacO* distances in *Synechocystis* may not only be due to a decreased local concentration of LacI at the primary repression site, but also due to a larger DNA-loop, which could exhibit a smaller hindrance for RNAP than a tighter one. Another similarity with *E. coli* is that the inter-*lacO* distances that represent maximum repression are not even multiples of 11 bp, corresponding to repression maxima at 55 and 66 bp, but one to two bp longer. This has been previously observed in vivo [23] and may be due to distortion of the intervening DNA sequence of the promoter by RNAP binding or the promoter DNA-sequence itself.

In *Synechocystis*, repression in the least optimal geometrical *lacO* positions, as represented by the expression peaks (Figure 5B), is very low even under non-induced conditions. As the P*trc*2O-library is identical to P*trc*1O-proximal, which is even less efficiently repressed, from the −35 element down to the TSS and the 5′ UTR, P*trc*2O-library promoters are expected to be similar to P*trc*1O-proximal in repression without DNA-looping. In fact, in the absence of LacI, the two highly repressed promoters P*trc*2O-2 and 13 were as or even more active than the fully induced P*trc*1O-prox (Figure 5A and B). Thus, P*trc*2O-library promoters whose *lacO* are out of phase are much less efficiently repressed in *Synechocystis* because of the weakly functioning proximal *lac* operator of P*trc*1O-proximal (Figure 5A and B), whereas their repression is stronger in *E. coli* for which P*trc*1O-proximal is more efficiently repressed (Figure 4A and B). As stated before, this difference in repression between *E. coli* NEB5α and *Synechocystis* for P*trc* and P*trc*1O-prox could be due to the about five times lower numbers of LacI/cell in the LacI-expressing *Synechocystis* strain. Thus, it is remarkable that repression of the P*trc*2O-library when the *lacO* are in phase is very efficient in *Synechocystis*, despite the weak primary proximal operator site and the lower levels of LacI/cell. In fact, the repression for geometrically optimal *lacO* positions is so efficient that IPTG induction almost fails to raise expression in *Synechocystis*, whereas it is possible to induce expression more in *E. coli*. Additionally, the repression troughs, which represent more optimal DNA-loop geometries, are wider in *Synechocystis* as compared to *E. coli*, and the expression peaks are narrower. Taken together, the stronger repression of an extremely leaky proximal *lacO*sym in optimal LacI-mediated DNA-loop configurations, the wider repression troughs, narrower expression peaks and the lower amounts of LacI/cell in *Synechocystis* as compared to *E. coli* have at least two possible implications. It implies that the *Synechocystis* RNAP is either more sensitive to DNA-looping than *E. coli* RNAP, and/or that *Synechocystis* chromatin DNA has lower in vivo torsion-resistance than *E. coli* chromatin DNA, lowering the barrier for forming twisted-DNA loops is the cyanobacterium.

## Conclusions

Artificial and minimal promoters orthogonal to cyanobacteria can be used for stable constitutive expression and pose an alternative to the use of native promoters. The proximal *lac* operator site of P*trc* and its sibling P*trc*1O-proximal is insufficient for efficient repression of transcription in *Synechocystis* under the amounts of LacI/cell tested here. We show that in vivo DNA-looping works as a mechanism of transcriptional regulation in cyanobacteria similarly as in *E. coli*. DNA-looping appears to be an efficient mechanism of repression in cyanobacteria, as promoters carrying the inefficient proximal *lacO* site could be more strongly repressed in combination with auxiliary distal *lac* operators in optimal phases in *Synechocystis* than in *E. coli*. The weak repression of promoters with only the proximal *lac* operator site in *Synechocystis* could simply be due to insufficient numbers of LacI/cell and target operator. Yet, as some P*trc*2O promoters are more efficiently repressed in *Synechocystis* than in *E. coli* despite the lower levels of Lac/cell in the cyanobacterium, we suggest that the repression differences could be due to previously observed differences in RNAP subunits. We tested for differences in the RNAP footprint using a proximal *lacO* promoter library but could not detect any discrepancy in pattern. The reasons for the more efficient repression of favorably-phased P*trc*2O promoters in *Synechocystis* than in *E. coli* are unclear. We speculate that it could be due to differences in the ease with which the chromatin DNA is looped or twisted, or differences in RNAP sensitivity to deformed DNA. As DNA-looping is very efficient for LacI-repression of promoters in *Synechocystis*, we suggest that an optimal design should incorporate this mechanism. Further, to avoid unpredictable expression levels of different genes due to interactions between coding sequences and 5’ UTRs on the mRNA level [56], future optimally repressed promoters would preferably involve DNA looping between core and distally located *lacO*. This allows for the exclusion of proximal *lac* operators from the 5' UTR and any possible effects they may have on the mRNA. Finally, the Ptrc2O (2) and (13) promoters display very high repression ratios in *Synechocystis* (about 400 and 170 times, respectively, Figure 5B), and could be used for genetic switches or gene expression systems that do not require subsequent induction. Alternatively, lower levels of LacI or unstable versions of LacI could be used to improve their inducibility.

## Methods

### DNA parts and sequences

All BioBrick parts, denoted by “BBa_”, and BioBrick plasmids were obtained from the iGEM Registry of Standard Biological Parts [34] (referred to as the iGEM Registry). Colony PCR on BioBrick plasmids was performed using the VF2 (BBa_G00100) and VR (BBa_G00101) flanking primers. Primer sequences (Table 2), promoter sequences (Table 1) and construct sequences (Additional file 1) are available.

**Table 2:** Primer DNA sequences

**Primer name**	**Sequence 5′ to 3′**
VF2 (BBa_G00100)	TGCCACCTGACGTCTAAGAA
VR (BBa_G00101)	ATTACCGCCTTTGAGTGAGC
RBS*-EYFP-BB-f1	CTTTCTAGAGTAGTGGAGGTTACTAGATGGTGAGCAAGGGCG
B0015-BB-r1	GAACTGCAGCGGCCGCTACTAGTATATAAACGCAGAAAGGCCC
j5_00029	GCGGCCGCTGCAGCCCGTAGAAAAGATCAAAGGATCTTCTTGAGA
j5_00030	GCCACGTAGGGGTCTCTAGAAGCGGCCGCGAATTCATGTGAGCAAAAGGCCAGCA
j5_00031	GCCGCTTCTAGAGACCCCTACGTGGCCGGCAATGGTCC
j5_00032	GGTGAAACTGACCGAACATAGGAGACTTTGGTGGGCTGGCCG
j5_00033	CCACCAAAGTCTCCTATGTTCGGTCAGTTTCACCTGATTTACG
j5_00079	CTGGTTTCACATTCACCATGATCAAACCTCCACTACTCTAGTAGCTAGCATTGTACCTAGGACTGAGCTAGCCATAAAGACAGTCATTCATCTTTCTGCCCCTCC
j5_00042	GTAGTGGAGGTTTGATCATGGTGAATGTGAAACCAGTAACGTTATACGATG
j5_00072	GTTCGTTAAGGCTTGATCTCTATTATTACTGCCCGCTTTCCAGTCGG
j5_00073	GGAAAGCGGGCAGTAATAATAGAGATCAAGCCTTAACGAACTAAGACCCC
j5_00088	CCCGATCAACTCGTGTCTGCTCCTCGGTTATGTTTTTAAGGTC
j5_00090	CGAGGAGCAGACACGAGTTGATCGGGCACGTAAGAGG
j5_00092	GCCATCCACTTCCTTGGTCTGACAGCTCGAGGCTTGG
j5_00093	GCTGTCAGACCAAGGAAGTGGATGGCCCCGTATTGC
j5_00039	CTTTGATCTTTTCTACGGGCTGCAGCGGCCGCTACTAGTAGGTCCCAAGTTTGTGCTGTGGC
slr0168-UR-f2	AAAGGGGACGAAGCCGCAGTTC
slr0168-DR-r2	ATCGCCGTGGTTAAATCCGTGG
P2OLib-r	TGAGCTACTAGTATGTGTGAAATTGTGAGCGCTCACAATTCCAC
P2OLib-f1O	TCAGAATTCGCGGCCGCTTCTAGAGCGAACGGTTCTGGCAAATATTC
P2OLib-f-2	TCAGAATTCGCGGCCGCTTCTAGAGAATTGTGAGCGCTCACAATTGACAATTAATCATCCGGCTCG
P2OLib-f-1	TCAGAATTCGCGGCCGCTTCTAGAGAATTGTGAGCGCTCACAATTTGACAATTAATCATCCGGCTCG
P2OLib-f0	TCAGAATTCGCGGCCGCTTCTAGAGAATTGTGAGCGCTCACAATTTTGACAATTAATCATCCGGCTC
P2OLib-f1	TCAGAATTCGCGGCCGCTTCTAGAGAATTGTGAGCGCTCACAATTGTTGACAATTAATCATCCGGC
P2OLib-f2	TCAGAATTCGCGGCCGCTTCTAGAGAATTGTGAGCGCTCACAATTTGTTGACAATTAATCATCCGG
P2OLib-f3	TCAGAATTCGCGGCCGCTTCTAGAGAATTGTGAGCGCTCACAATTCTGTTGACAATTAATCATCCGG
P2OLib-f4	TCAGAATTCGCGGCCGCTTCTAGAGAATTGTGAGCGCTCACAATTGCTGTTGACAATTAATCATCCGG
P2OLib-f5	TCAGAATTCGCGGCCGCTTCTAGAGAATTGTGAGCGCTCACAATTAGCTGTTGACAATTAATCATCCG
P2OLib-f6	TCAGAATTCGCGGCCGCTTCTAGAGAATTGTGAGCGCTCACAATTGAGCTGTTGACAATTAATCATCCG
P2OLib-f7	TCAGAATTCGCGGCCGCTTCTAGAGAATTGTGAGCGCTCACAATTTGAGCTGTTGACAATTAATCATCC
P2OLib-f8	TCAGAATTCGCGGCCGCTTCTAGAGAATTGTGAGCGCTCACAATTATGAGCTGTTGACAATTAATCATCC
P2OLib-f9	TCAGAATTCGCGGCCGCTTCTAGAGAATTGTGAGCGCTCACAATTAATGAGCTGTTGACAATTAATCATCC
P2OLib-f10	TCAGAATTCGCGGCCGCTTCTAGAGAATTGTGAGCGCTCACAATTAAATGAGCTGTTGACAATTAATCATC
P2OLib-f11	TCAGAATTCGCGGCCGCTTCTAGAGAATTGTGAGCGCTCACAATTGAAATGAGCTGTTGACAATTAATCATC
P2OLib-f12	TCAGAATTCGCGGCCGCTTCTAGAGAATTGTGAGCGCTCACAATTTGAAATGAGCTGTTGACAATTAATC
P2OLib-f13	TCAGAATTCGCGGCCGCTTCTAGAGAATTGTGAGCGCTCACAATTCTGAAATGAGCTGTTGACAATTAATC
P2OLib-f14	TCAGAATTCGCGGCCGCTTCTAGAGAATTGTGAGCGCTCACAATTTCTGAAATGAGCTGTTGACAATTAATC
P2OLib-f15	TCAGAATTCGCGGCCGCTTCTAGAGAATTGTGAGCGCTCACAATTTTCTGAAATGAGCTGTTGACAATTA
P2OLib-f16	TCAGAATTCGCGGCCGCTTCTAGAGAATTGTGAGCGCTCACAATTATTCTGAAATGAGCTGTTGACAATT
P2OLib-f17	TCAGAATTCGCGGCCGCTTCTAGAGAATTGTGAGCGCTCACAATTTATTCTGAAATGAGCTGTTGACAATT
P2OLib-f18	TCAGAATTCGCGGCCGCTTCTAGAGAATTGTGAGCGCTCACAATTATATTCTGAAATGAGCTGTTGACAATT
P2OLib-f19	TCAGAATTCGCGGCCGCTTCTAGAGAATTGTGAGCGCTCACAATTAATATTCTGAAATGAGCTGTTGACAA
P2OLib-f20	TCAGAATTCGCGGCCGCTTCTAGAGAATTGTGAGCGCTCACAATTAAATATTCTGAAATGAGCTGTTGACA
P2OLib-f21	TCAGAATTCGCGGCCGCTTCTAGAGAATTGTGAGCGCTCACAATTCAAATATTCTGAAATGAGCTGTTGAC
P1Od-f1	AAGTCTAGAGAATTGTGAGCGCTCACAATTTTGACAATTAATCATCCGGCTCGTATAATGTGTGGAATCACACATACTAGAGTAGTGGAGGTTACTAGATGG
P1Oc-f1	AAGTCTAGAGAAATATTCTGAAATGAGCTGTTGACAATTGTGAGCGCTCACAATATAATGTGTGGAATCACACATACTAGAGTAGTGGAGGTTACTAGATGG
PA1lacO1-FP-f1	AAGTCTAGAGAAAGAGTGTTGACTTGTGAGCGGATAACAATGATACTTAGATTCAATTGTGAGCGGATAACAATTTCACACATACTAGAGTAGTGGAGGTTACTAGATGG
PtrcOs-prox-8-f1	TTCTCTAGAGTTGACAATTAATCATCCGGCTCGTATAATGTGTAATTGTGAGCGCTCACAATTTACTAGAGTAGTGGAGGTTACTAGATGG
PtrcOs-prox-9-f1	TTCTCTAGAGTTGACAATTAATCATCCGGCTCGTATAATGTGTGAATTGTGAGCGCTCACAATTTACTAGAGTAGTGGAGGTTACTAGATGG
PtrcOs-prox-10-f1	TTCTCTAGAGTTGACAATTAATCATCCGGCTCGTATAATGTGTGGAATTGTGAGCGCTCACAATTTACTAGAGTAGTGGAGGTTACTAGATGG
PtrcOs-prox-11-f1	TTCTCTAGAGTTGACAATTAATCATCCGGCTCGTATAATGTGTGGAAATTGTGAGCGCTCACAATTTACTAGAGTAGTGGAGGTTACTAGATGG
PtrcOs-prox-12-f1	TTCTCTAGAGTTGACAATTAATCATCCGGCTCGTATAATGTGTGGAGAATTGTGAGCGCTCACAATTTACTAGAGTAGTGGAGGTTACTAGATGG
PtrcOs-prox-13-f1	TTCTCTAGAGTTGACAATTAATCATCCGGCTCGTATAATGTGTGGAGTAATTGTGAGCGCTCACAATTTACTAGAGTAGTGGAGGTTACTAGATGG
PtrcOs-prox-14-f1	TTCTCTAGAGTTGACAATTAATCATCCGGCTCGTATAATGTGTGGAGTCAATTGTGAGCGCTCACAATTTACTAGAGTAGTGGAGGTTACTAGATGG
PtrcOs-prox-15-f1	TTCTCTAGAGTTGACAATTAATCATCCGGCTCGTATAATGTGTGGAGTCGAATTGTGAGCGCTCACAATTTACTAGAGTAGTGGAGGTTACTAGATGG
PtrcOs-prox-16-f1	TTCTCTAGAGTTGACAATTAATCATCCGGCTCGTATAATGTGTGGAGTCGTAATTGTGAGCGCTCACAATTTACTAGAGTAGTGGAGGTTACTAGATGG
PtrcOs-prox-17-f1	TTCTCTAGAGTTGACAATTAATCATCCGGCTCGTATAATGTGTGGAGTCGTCAATTGTGAGCGCTCACAATTTACTAGAGTAGTGGAGGTTACTAGATGG
PtrcOs-prox-18-f1	TTCTCTAGAGTTGACAATTAATCATCCGGCTCGTATAATGTGTGGAGTCGTCCAATTGTGAGCGCTCACAATTTACTAGAGTAGTGGAGGTTACTAGATGG
PtrcOs-prox-20-f1	TTCTCTAGAGTTGACAATTAATCATCCGGCTCGTATAATGTGTGGAGTCGTCCAGAATTGTGAGCGCTCACAATTTACTAGAGTAGTGGAGGTTACTAGATGG
PtrcOs-prox-22-f1	TTCTCTAGAGTTGACAATTAATCATCCGGCTCGTATAATGTGTGGAGTCGTCCAGACAATTGTGAGCGCTCACAATTTACTAGAGTAGTGGAGGTTACTAGATGG
PtrcOs-prox-24-f1	TTCTCTAGAGTTGACAATTAATCATCCGGCTCGTATAATGTGTGGAGTCGTCCAGACTCAATTGTGAGCGCTCACAATTTACTAGAGTAGTGGAGGTTACTAGATGG

### Strains and culture conditions

*Escherichia coli* strain NEB5α F’ *I*^*q*^ (*lacI*^*q*^ strain, New England Biolabs, referred to as *E. coli*) was used for cloning and promoter characterization of the constitutive, control and P*trc*2O-library promoters. *E. coli* strain DH5αZ1 (carries two *lacI*^*q*^ genes at the *attB* locus, Expressys) was used for cloning and promoter characterization of the proximal *lacO*-library. The *lacI*^*q*^ gene is a promoter-mutated version of the wild-type *lacI* gene that causes about ten times as much LacI to be expressed as compared with the wild-type strain [57]. The strains were cultured in LB medium at 37°C shaking at 225 rpm, or on LB-agar plates, and were supplemented with 25 μg/mL kanamycin for pPMQAK1 [25] [iGEM Registry: BBa_J153000, Genbank: GU933126, Addgene: 26052] constructs, 50 μg/mL kanamycin for pSB1AK3 [34] constructs or 35 μg/mL chloramphenicol for the *Synechocystis lacI* or Chloramphenicol resistance-only recombination vectors.

*Synechocystis* PCC 6803 (the glucose tolerant strain ATCC 27184, referred to as *Synechocystis*) was cultured in BG11 medium [58], or on BG11-agar plates, supplemented with 50 μg/mL kanamycin for pPMQAK1 constructs. The *Synechocystis* engineered strain expressing LacI and the strain carrying only Chloramphenicol resistance were cultured in BG11 supplemented with 25 μg/mL chloramphenicol, or both 25 μg/mL kanamycin and 25 μg/mL chloramphenicol when containing pPMQAK1 constructs. All strains were grown at 30°C under continuous white light of 20-50 μmol photons m^-2^ s^-1^ shaking at 120 rpm unless otherwise stated.

### Construction and characterization of constitutive promoters in *Synechocystis*

Our selection criteria for promoters expressing the Lac repressor in *Synechocystis* were minimal constitutive promoters orthogonal to *Synechocystis*. For this purpose, we selected several members of an artificial σ-70 promoter library spanning a wide range of activities in *E. coli* for characterization in *Synechocystis* (BBa_J23100, BBa_J23101, BBa_J23102, BBa_J23105, BBa_J23106, BBa_J23109, BBa_J23113 and BBa_J23114, from the iGEM Registry’s Anderson collection [34]). For comparison, we selected three promoters commonly used for engineered expression in *Synechocystis;* the nitrate reductase promoter P*nirA* (synthesized by Epoch Biolabs Inc.), the plastocyanin promoter P*petE* (BBa_K273019, from iGEM-team Uppsala-Sweden 2009) and the RNase P subunit B promoter P*rnpB*[25]. An EYFP reporter cassette containing RBS*, a ribosome binding site that is highly active in both *E. coli* and *Synechocystis*[4], EYFP itself (BBa_E0030) and a double transcriptional terminator (BBa_B0015) was constructed by PCR using the Phusion Hot-Start II High-Fidelity DNA polymerase (Thermo Scientific, referred to as Phusion) with primers RBS*-EYFP-BB-f1, B0015-BB-r1 and another EYFP reporter cassette as template (BBa_E0430) according to the manufacturer’s instructions. The promoters were cloned together with the EYFP reporter cassette into pSB1AK3 using BioBrick 3A-assembly [59], subcloned into pPMQAK1 and confirmed by sequencing. The pPMQAK1-carried constructs plus an empty pPMQAK1 vector control plasmid [25] were transferred to *Synechocystis* through conjugation according to a previously published procedure [4] using *E. coli* strain HB101 with pRL443 [60] as the conjugal plasmid and BG11 plates with 50 μg/mL kanamycin for selection. Single *Synechocystis* colonies were grown in flat-bottomed tissue culture 6-well plates (Sarstedt) with lids taped shut with Micropore tape in 5 mL BG11 supplemented with 50 μg/mL kanamycin at ca 50 μmol photons m^-2^ s^-1^ (protecting cultures from light with white paper the first days) for about two weeks (corresponding to a final absorbance at 750 nm (Abs750) of 0.6-1.3 as measured in translucent 96-well tissue culture plates using a Chameleon V Microplate Reader (Hidex, referred to as plate reader)). The presence of the promoter-reporter plasmids was confirmed with colony PCR and the cultures were inspected for the absence of *E. coli* using microscopy. To produce promoter characterization seed cultures in the same growth phase, all cultures were diluted to Abs750 = 0.01 into E-flasks with 25 mL medium and grown for four days in the same conditions as before, reaching a final Abs750 of 0.3-0.45. The seed cultures were used to inoculate measurement cultures to Abs750 = 0.01, which were split into six replicate cultures each of 5 mL in 6-well plates with sealed lids. The measurement cultures were grown for 42 hours whereupon samples were taken for plate reader measurements of Abs750 and EYFP fluorescence using 485 nm emission and 535 nm excitation filters. Black 96-well plates with clear flat bottom (BD Falcon) were used to eliminate well-to-well fluorescence cross-talk. After subtracting medium and instrument background, fluorescence per cell values were calculated by normalizing fluorescence values with Abs750 values, averaging over the six replicates and estimating the standard error of the mean. Finally, specific fluorescence per cell values for each promoter construct was obtained by subtracting cellular background fluorescence from the empty pPMQAK1 control culture.

### Construction of LacI-expressing and Chloramphenicol resistance-only strains of *Synechocystis*

As we have previously found that the P*rnpB* promoter in combination with the BBa_B0034 RBS express amounts of LacI strongly repressing P*trc*2O when both constructs are carried on the pPMQAK1 plasmid [25], we selected the weaker (as characterized in this study) and synthetic BBa_J23114 promoter in combination with RBS* [4] to express LacI from the chromosome. For insertion of the LacI-expressing cassette into the chromosome we selected *slr0168*, a previously used neutral site [61,62] that displays very low transcript levels [37]. The LacI-expression cassette, which is flanked by *slr0168* insertion sequences, consists of a forward terminator to insulate the cassette from potentially incoming chromosomal RNAP (*rnpB*_T1 from *E. coli*), BBa_J23114 in combination with RBS* to drive LacI expression, the *lacI* CDS, another forward terminator (*ilvGEDA*_T from *E. coli*) and a chloramphenicol resistance cassette (CmR) derived from pSB1AC3 (iGEM Registry). The LacI-expression cassette together with a pMB1 replicon for replication in *E. coli* were assembled using one-step isothermal assembly [63] using the j5 DNA assembly design automation software [64] for primer design. Specifically, primers j5_00029 and j5_00030 were used to PCR the pMB1 replicon using pBluescript II SK + (Stratagene) as template, j5_00031 and j5_00032 to PCR the *slr0168* upstream recombination recombination sequence using *Synechocystis* genomic DNA as template, j5_00033 and j5_00079 to PCR *rnpB*_T1 and the promoter-RBS region using *rnpB*_T1 synthetic DNA (Genscript) as template, j5_00042 and j5_00072 to PCR *lacI* using *E. coli* strain JM107 genomic DNA as template, j5_00073 and j5_00088 to PCR *ilvGEDA*_T using synthetic DNA (Genscript) as template, j5_00090 and j5_00092 to PCR CmR using pSB1AC3 as template and finally j5_00093 and j5_00039 to PCR the *slr0168* downstream recombination sequence using *Synechocystis* genomic DNA as template. The upstream recombination and the *rnpB*_T1-promoter-RBS fragments, and the *ilvGEDA*_T and CmR fragments, were spliced together by overlap extension (SOE) before one-step isothermal assembly. The chloramphenicol resistance-only construct was constructed analogously to the LacI-expressing construct, but all elements except the recombination sites, the second forward terminator (*ilvGEDA*_T) and the chloramphenicol resistance cassette were excluded. The sequences of both constructs are available in Additional file 1. All PCR and SOE reactions were made using Phusion. The resulting pMB1-replicated LacI-expression cassette and the chloramphenicol resistance-only construct were sequence-confirmed, transferred to *Synechocystis* by natural transformation and inserted into the genomic *slr0168* target site by homologous recombination according to previously published procedures [4]. Mutants were selected on BG11 plates supplemented with 10 μg/mL chloramphenicol and single colonies were streaked on BG11 25 μg/mL chloramphenicol plates twice to allow for full segregation. Finally, the fully segregated mutants were confirmed with colony PCR and sequencing of the insertion site using primers slr0168-UR-f2 and slr0168-DR-r2.

To confirm and quantify the expression of LacI in the LacI-expressing strain of *Synechocystis*, a quantitative western blot was performed with anti-LacI polyclonal antibodies (PAB10255, raised in rabbit, Abnova) and secondary HRP-conjugated anti-rabbit antibodies (Immun-Star GAR-HRP conjugate, #170-5046, BioRad). Briefly, a known number of cells from three biological replicates of DH5αZ1, two replicates of NEB5α, and three replicates of the LacI-expressing strain of *Synechocystis* carrying different promoter reporter constructs on pPMQAK1, were denatured in reducing SDS sample buffer for 5 mins at 95°C. The samples were run on a PAGE gel (any-kD precast 8-well gels, BioRad) and blotted to membranes (TransBlot turbo, midi transfer packs with PVDF membranes, BioRad). Western blotting was performed according to the manufacturer’s instruction with the exception that the primary LacI antibody was hybridized over night. The blot was developed using HRP chemiluminescence (Immun-Star HRP substrate kit, BioRad) and the band intensities were quantified using QuantityOne software (BioRad). The average band intensities were normalized with the number of loaded cells to get relative values of LacI/cell for the different strains. For an absolute value of LacI tetramers/cell for all strains, a previous quantification of 3000 tetramers of LacI/cell for DH5αZ1 [36] was used as a standard.

### Construction and characterization of the P*trc-*derived promoter library in *E. coli* and *Synechocystis*

The P*trc*2O-library and P*trc*1O-prox promoters were made with Phusion PCR using the reverse primer P2OLib-r to exchange *lacO*1 of the original P*trc*[24] and P*trc*2O [25] (here referred to as P*trc*2O-orig) to the symmetric *lacO*sym operator. Different forward primers adding another *lacO*sym upstream the −35 box with spacers of different length in between (primers P2OLib-f-n for the P*trc*2O-library, where n is a numeral representing the spacer length ranging from −2 to 21, and primer P2OLib-f1O for P*trc*1O-prox that does not include a second *lacO*sym) were used, together with P*trc*2O-orig as PCR template. The promoter PCR products were assembled with the same EYFP reporter cassette as the constitutive promoters into pPMQAK1 using 3A-assembly. The single *lacO*sym control promoters P*trc*1O-dist, with *lacO*sym immediately distal to the −35 box, and P*trc*1O-core, with a slightly truncated *lacO*sym in the core region between the −35 and the −10 boxes, and the PA1*lacO*-1 promoter [36], were made with Phusion PCR using the different forward primers P1Od-f1 (for P*trc*1O-dist), P1Oc-f1 (for P*trc*1O-core) and PA1lacO1-FP-f1 (for PA1*lacO*-1) and the same B0015-BB-r1 reverse primer using the EYFP reporter cassette as template. These PCR products were also cloned into pPMQAK1 using the BioBrick cloning site. After sequence confirmation using the VF2 and VR primers all promoters were first characterized in *E. coli* and then transferred to the LacI-expressing *Synechocystis* strain for subsequent characterization.

For characterization of the P*trc*-derived promoter library in *E. coli* the following protocol was repeated at three different days, producing a total of six biological measurement replicates. Two overnight cultures for each promoter construct were inoculated from frozen glycerol stocks in 2 mL LB supplemented with 25 μg/mL kanamycin in 13 mL growth tubes (Sarstedt) and grown at 37°C shaking at 225 rpm until all cultures had reached stationary phase (approximately 22 hours). The overnight cultures were put into ice-water to ensure that both the negative and positive Isopropyl β-D-1-thiogalactopyranoside (IPTG) cultures had the same starting conditions. For each culture, 1 μl was transferred to a 96-well tissue culture plate pre-loaded with 99 μl LB supplemented with 25 μg/mL kanamycin and either 0 or 1 mM IPTG for the non-induced plate and induced plate, respectively. Only the middle 60 wells were used as the evaporation rate was observed to be higher for the outermost wells. The plates were sealed with Breathe-Easy sealing membranes (Sigma-Aldrich) and incubated shaking at level 8 on a Delfia plate shaker (Wallac) at 37°C for 7 hours. Samples were taken and measured in black 96-well plates with clear flat bottom in the same way as for the constitutive promoters in *Synechocystis*, but Abs595 was used instead of Abs750.

For characterization in *Synechocystis* the P*trc*-derived promoter library was transferred to the LacI-expressing *Synechocystis* strain by conjugation as was done for the constitutive promoters to wild-type *Synechocystis*, but BG11 plates with 25 μg/mL kanamycin and 25 μg/mL chloramphenicol was used for selection. Two colonies for each promoter construct were inoculated into ca 2 mL BG11 with 25 μg/mL kanamycin and 25 μg/mL chloramphenicol in 13 mL growth tubes shaking in low light conditions (ca 5 μmol photons m^-2^ s^-1^) for ca three weeks (reaching a final Abs750 ranging from 1 to 3.5). To ensure that the seed cultures are in the same growth phase all cultures were diluted to Abs750 = 0.05 and grown under the same conditions as the parent cultures for 44 hours. For measurements, all cultures were diluted once more in the 13 mL growth tubes to Abs750 = 0.05 and 200 μl of each was loaded into the middle 32 wells of 96-well tissue culture plates, where the induction plates were pre-loaded with 1 μl 200 mM IPTG, and mixed by pipetting. The plate measurement cultures were grown with the lids on shaking at level 4 on a Delfia plateshaker at 30°C in white light of ca 50 μmol photons m^-2^ s^-1^ for 46 hours, after which samples were taken for measurements. The cultures in the 13 mL growth tubes were put back in the same low-light conditions immediately after starting the first measurement cultures and after two days they were used to start new measurement cultures, leading to a total of four biological replicates for all promoter constructs. The second round of measurement cultures were produced in the same way as the first one, but the cultures were diluted to Abs750 = 0.05 directly in the 96-well plates, and grown under the same conditions for the same time. Samples were taken and measured in black 96-well plates with clear flat bottom in the same way as for the constitutive promoters in *Synechocystis*, but averages and standard errors of the mean were calculated for four replicates instead of six.

The control promoter constructs BBa_J23101, P*trc*1O-core, PA1*lacO*-1 and the P*trc*2O-library constructs P*trc*2O-2, 13 and 18 were transferred into the chloramphenicol resistance-only strain of *Synechocystis* to enable characterization of these promoters without the presence of LacI. Conjugation and characterization was carried out in a similar way as for the LacI-expressing *Synechocystis* strain, but the measurement cultures were inoculated into 1 mL medium in 13 mL growth tubes instead of 96-well plates and grown at 10-15 μmol photons m^-2^ s^-1^. Only one round of measurement cultures were produced (yielding two biological replicates). The cultures were measured in the same way as for the LacI-expressing *Synechocystis* strain but averages and standard errors of the mean were calculated for two replicates instead of four.

### Construction and characterization of the proximal *lacO*-library in *E. coli* DH5αZ1 and *Synechocystis*

The proximal *lacO* promoter library was made with Phusion PCR using reverse primer B0015-BB-r1 together with the forward primers PtrcOs-prox-n-f1. The letter n is a numeral ranging from 8-18 in steps of one and from 18-24 in steps of two, representing the distance in bp between the center of a proximal *lacOsym* to the TSS of a P*trc* core promoter (Table 1). The PCR was performed using a strongly enhanced green fluorescent protein (SGFP2) [65] codon-optimized for *E. coli* (DNA 2.0, a gift from Erik Gullberg) reporter construct consisting of RBS*-SGFP2-BBa_B0015 (Additional file 1) as template. The PCR products were cloned into pPMQAK1 using the BioBrick cloning site. After sequence confirmation using the VF2 and VR primers all promoters were characterized in *E. coli* DH5αZ1 and transferred to both the LacI-expressing *Synechocystis* strain and the chloramphenicol resistance-only strain for subsequent characterization.

Characterization in *E. coli* DH5αZ1 was done similarly as for the P*trc*-derived promoter library in NEB5α but six biological replicates were characterized divided on two different days. The cultures were grown directly in black 96-well plates with clear flat bottom diluted 1:200 in 200 μl medium supplemented with water or 2 mM IPTG, shaking at level 7 on the plate shaker. The cultures were measured without lids at several time-points after the start of the experiment (approximately 0, 2.4, 3.8, 4.2, 4.7, 5.1 and 5.5 hours after dilution and induction) to observe for possible fluorescence/absorbance steady states and the temporal development of the induction ratios. The induction ratios and their estimated standard errors were calculated from the ratio of induced/non-induced fluorescence/absorbance data.

Conjugation to and characterization in the LacI-expressing *Synechocystis* strain and the chloramphenicol resistance-only strain was done similarly as for the P*trc*-derived promoter library in the LacI-expressing *Synechocystis* strain. The measurement cultures were inoculated into 1 mL medium supplemented with water or 2 mM IPTG in 13 mL growth tubes instead of 96-well plates, and grown at 10-15 μmol photons m^-2^ s^-1^. Only one round of measurement cultures were produced (yielding two biological replicates). The cultures were measured in the same way as the P*trc*-derived promoter library in the LacI-expressing *Synechocystis* strain. The induction ratios and their estimated standard errors were calculated from the ratio of induced/non-induced fluorescence/absorbance data for the LacI-expressing *Synechocystis* strain and the repression ratios and their estimated standard errors were calculated from the ratio of the fluorescence/absorbance data of the chloramphenicol resistance-only strain/the data from the LacI-expressing strain.

## Competing interests

The authors declare that they have no competing interests.

## Author’s contributions

DC, TH and PL conceived of the research; DC designed the study and performed the experiments; DC and PL analyzed the data; DC and PL wrote the manuscript. All authors approved of the final manuscript.

## Supplementary Material

Additional file 1 DNA sequences of the EYFP reporter, the SGFP2 reporter, the LacI-expression cassette and the chloramphenicol resistance-only construct. Click here for file
