# Supplementary material for: Design and analysis of LacI-repressed promoters and DNA-looping in a cyanobacterium
Source: J Biol Eng. 2014 Jan 27;8:4. doi: 10.1186/1754-1611-8-4 (PMC3922697; doi:10.1186/1754-1611-8-4)
Supplement: Additional file 1 — DNA sequences of the EYFP reporter, the SGFP2 reporter, the LacI-expression cassette and the chloramphenicol resistance-only construct. [file 1754-1611-8-4-S1.docx]

Additional file 1 – Construct DNA sequences

Design and Analysis of LacI-repressed Promoters and DNA-looping in a Cyanobacterium

Daniel Camsund1, Thorsten Heidorn2 and Peter Lindblad1*

1 Microbial Chemistry, Department of Chemistry - Ångström Laboratory, Uppsala University, P.O. Box 523, SE-75120, Uppsala, Sweden.

2 Bioforsk, Frederik A. Dahls vei 20, 1432 Ås, Norway.

* Corresponding author: [peter.lindblad@kemi.uu.se](mailto:peter.lindblad@kemi.uu.se).

**Contents:**

DNA sequence of the enhanced yellow fluorescent protein (EYFP) reporter construct

DNA sequence of the green fluorescent protein (SGFP2) reporter construct

DNA sequence of the LacI-expression plasmid for insertion into the *Synechocystis* PCC 6803 chromosome

DNA sequence of the chloramphenicol resistance-only plasmid for insertion into the *Synechocystis* PCC 6803 chromosome

**DNA sequence of the enhanced yellow fluorescent protein (EYFP) reporter construct**

Key: BioBrick prefix, Ribosome binding site (RBS*), Reporter (EYFP), Double transcriptional terminator (BBa_B0015) , BioBrick suffix

GAATTCGCGGCCGCTTCTAGAGTAGTGGAGGTTACTAGATGGTGAGCAAGGGCGAGGAGCTGTTCACCGGGGTGGTGCCCATCCTGGTCGAGCTGGACGGCGACGTAAACGGCCACAAGTTCAGCGTGTCCGGCGAGGGCGAGGGCGATGCCACCTACGGCAAGCTGACCCTGAAGTTCATCTGCACCACCGGCAAGCTGCCCGTGCCCTGGCCCACCCTCGTGACCACCTTCGGCTACGGCCTGCAATGCTTCGCCCGCTACCCCGACCACATGAAGCTGCACGACTTCTTCAAGTCCGCCATGCCCGAAGGCTACGTCCAGGAGCGCACCATCTTCTTCAAGGACGACGGCAACTACAAGACCCGCGCCGAGGTGAAGTTCGAGGGCGACACCCTGGTGAACCGCATCGAGCTGAAGGGCATCGACTTCAAGGAGGACGGCAACATCCTGGGGCACAAGCTGGAGTACAACTACAACAGCCACAACGTCTATATCATGGCCGACAAGCAGAAGAACGGCATCAAGGTGAACTTCAAGATCCGCCACAACATCGAGGACGGCAGCGTGCAGCTCGCCGACCACTACCAGCAGAACACCCCCATCGGCGACGGCCCCGTGCTGCTGCCCGACAACCACTACCTGAGCTACCAGTCCGCCCTGAGCAAAGACCCCAACGAGAAGCGCGATCACATGGTCCTGCTGGAGTTCGTGACCGCCGCCGGGATCACTCTCGGCATGGACGAGCTGTACAAGTAATAATACTAGAGCCAGGCATCAAATAAAACGAAAGGCTCAGTCGAAAGACTGGGCCTTTCGTTTTATCTGTTGTTTGTCGGTGAACGCTCTCTACTAGAGTCACACTGGCTCACCTTCGGGTGGGCCTTTCTGCGTTTATATACTAGTAGCGGCCGCTGCAG

**DNA sequence of the green fluorescent protein (SGFP2) reporter construct**

Key: Ribosome binding site (RBS*), Reporter (SGFP2), Double transcriptional terminator (BBa_B0015), BioBrick suffix

<Promoter>TACTAGAGTAGTGG**A**GGTTACTAGATGGTTAGCAAGGGCGAAGAACTTTTTACAGGCGTAGTACCGATCTTAGTTGAATTAGACGGCGACGTTAACGGTCATAAGTTTAGCGTGAGCGGTGAGGGTGAAGGTGACGCAACTTACGGCAAGCTGACCCTGAAGTTCATTTGCACGACGGGTAAGCTGCCGGTCCCGTGGCCTACCCTGGTCACGACCTTGACTTATGGCGTTCAGTGTTTCGCGCGTTATCCGGACCACATGAAACAACACGATTTCTTTAAGAGCGCGATGCCAGAAGGCTATGTGCAGGAGCGTACGATCTTTTTCAAAGACGACGGTAACTACAAGACGCGTGCCGAAGTCAAATTCGAAGGCGACACCCTGGTGAATCGCATTGAGCTGAAGGGTATTGATTTCAAAGAGGATGGCAATATCCTGGGTCACAAGCTGGAGTACAATTACAATTCCCACAACGTTTACATCACCGCAGATAAACAGAAAAATGGCATCAAAGCGAATTTCAAAATCCGTCACAACATTGAGGACGGTGGTGTCCAACTGGCGGATCATTACCAGCAAAACACCCCGATTGGTGACGGTCCGGTCCTGTTGCCGGATAACCATTATCTGTCTACGCAAAGCAAACTGAGCAAAGATCCGAACGAGAAGCGCGACCACATGGTGCTGCTGGAGTTTGTGACCGCTGCCGGTATTACCCTGGGTATGGATGAGCTGTATAAATAATAATACTAGAGCCAGGCATCAAATAAAACGAAAGGCTCAGTCGAAAGACTGGGCCTTTCGTTTTATCTGTTGTTTGTCGGTGAACGCTCTCTACTAGAGTCACACTGGCTCACCTTCGGGTGGGCCTTTCTGCGTTTATATACTAGTAGCGGCCGCTGCAG

**DNA sequence of the LacI-expression plasmid for insertion into the *Synechocystis* PCC 6803 chromosome**

Key: pMB1-replicon, BioBrick prefix, *slr0168* recombination sequence, *rnpB*_T1, BBa_J23114 promoter and RBS*, *lacI* CDS, *ilvGEDA*_T, Predicted promoter and RBS, *cat* CDS (CmR), Predicted terminator, *slr0168* recombination sequence, BioBrick suffix

CCCGTAGAAAAGATCAAAGGATCTTCTTGAGATCCTTTTTTTCTGCGCGTAATCTGCTGCTTGCAAACAAAAAAACCACCGCTACCAGCGGTGGTTTGTTTGCCGGATCAAGAGCTACCAACTCTTTTTCCGAAGGTAACTGGCTTCAGCAGAGCGCAGATACCAAATACTGTCCTTCTAGTGTAGCCGTAGTTAGGCCACCACTTCAAGAACTCTGTAGCACCGCCTACATACCTCGCTCTGCTAATCCTGTTACCAGTGGCTGCTGCCAGTGGCGATAAGTCGTGTCTTACCGGGTTGGACTCAAGACGATAGTTACCGGATAAGGCGCAGCGGTCGGGCTGAACGGGGGGTTCGTGCACACAGCCCAGCTTGGAGCGAACGACCTACACCGAACTGAGATACCTACAGCGTGAGCTATGAGAAAGCGCCACGCTTCCCGAAGGGAGAAAGGCGGACAGGTATCCGGTAAGCGGCAGGGTCGGAACAGGAGAGCGCACGAGGGAGCTTCCAGGGGGAAACGCCTGGTATCTTTATAGTCCTGTCGGGTTTCGCCACCTCTGACTTGAGCGTCGATTTTTGTGATGCTCGTCAGGGGGGCGGAGCCTATGGAAAAACGCCAGCAACGCGGCCTTTTTACGGTTCCTGGCCTTTTGCTGGCCTTTTGCTCACATGAATTCGCGGCCGCTTCTAGAGACCCCTACGTGGCCGGCAATGGTCCCAAAATTGTCGCCGCTAAGTTAGACCGCTTCAGTGACCTGGGGGAAGGGGCTCCCCTCTGGTTAGCCACCAATCAAAATAACAGTGGCGGGGATTTATATGGAGACCAAGCCCAATTTCGTTTGCGAATTTACACCAGCGCCGGTTTTTCCCCCGATGGCATTGCCAGTTTACTACCCACAGAATTTGAACGGTATTTTCAACTCCAAGCGGAAGATATTACGGGACGGACAGTTATCCTAACCCAAACTGGTGTTGATTATGAAATTCCCGGCTTTGGTCTGGTGCAGGTGTTGGGGCTGGCGGATTTGGCCGGGGTTCAGGACAGCTATGACCTGACTTACATCGAAGATCATGACAACTATTACGACATTATCCTCAAAGGGGACGAAGCCGCAGTTCGCCAAATTAAGAGGGTTGCTTTGCCCTCCGAAGGGGATTATTCGGCGGTTTATAATCCCGGTGGCCCCGGCAATGATCCAGAGAATGGTCCCCCAGGGCCCTTTACTGTGTCCAGTAGTCCCCAGGTAATTAAGGTAACGGATACCATCGGCCAGCCCACCAAAGTCTCCTATGTTCGGTCAGTTTCACCTGATTTACGTAAAAACCCGCTTCGGCGGGTTTTTGCTTTTGGAGGGGCAGAAAGATGAATGACTGTCTTTATGGCTAGCTCAGTCCTAGGTACAATGCTAGCTACTAGAGTAGTGGAGGTTTGATCATGGTGAATGTGAAACCAGTAACGTTATACGATGTCGCAGAGTATGCCGGTGTCTCTTATCAGACCGTTTCCCGCGTGGTGAACCAGGCCAGCCACGTTTCTGCGAAAACGCGGGAAAAAGTGGAAGCGGCGATGGCGGAGCTGAATTACATTCCCAACCGCGTGGCACAACAACTGGCGGGCAAACAGTCGTTGCTGATTGGCGTTGCCACCTCCAGTCTGGCCCTGCACGCGCCGTCGCAAATTGTCGCGGCGATTAAATCTCGCGCCGATCAACTGGGTGCCAGCGTGGTGGTGTCGATGGTAGAACGAAGCGGCGTCGAAGCCTGTAAAGCGGCGGTGCACAATCTTCTCGCGCAACGCGTCAGTGGGCTGATCATTAACTATCCGCTGGATGACCAGGATGCCATTGCTGTGGAAGCTGCCTGCACTAATGTTCCGGCGTTATTTCTTGATGTCTCTGACCAGACACCCATCAACAGTATTATTTTCTCCCATGAAGACGGTACGCGACTGGGCGTGGAGCATCTGGTCGCATTGGGTCACCAGCAAATCGCGCTGTTAGCGGGCCCATTAAGTTCTGTCTCGGCGCGTCTGCGTCTGGCTGGCTGGCATAAATATCTCACTCGCAATCAAATTCAGCCGATAGCGGAACGGGAAGGCGACTGGAGTGCCATGTCCGGTTTTCAACAAACCATGCAAATGCTGAATGAGGGCATCGTTCCCACTGCGATGCTGGTTGCCAACGATCAGATGGCGCTGGGCGCAATGCGCGCCATTACCGAGTCCGGGCTGCGCGTTGGTGCGGATATCTCGGTAGTGGGATACGACGATACCGAAGACAGCTCATGTTATATCCCGCCGTTAACCACCATCAAACAGGATTTTCGCCTGCTGGGGCAAACCAGCGTGGACCGCTTGCTGCAACTCTCTCAGGGCCAGGCGGTGAAGGGCAATCAGCTGTTGCCCGTCTCACTGGTGAAAAGAAAAACCACCCTGGCGCCCAATACGCAAACCGCCTCTCCCCGCGCGTTGGCCGATTCATTAATGCAGCTGGCACGACAGGTTTCCCGACTGGAAAGCGGGCAGTAATAATAGAGATCAAGCCTTAACGAACTAAGACCCCCGCACCGAAAGGTCCGGGGGTTTTTTTTGACCTTAAAAACATAACCGAGGAGCAGACACGAGTTGATCGGGCACGTAAGAGGTTCCAACTTTCACCATAATGAAATAAGATCACTACCGGGCGTATTTTTTGAGTTATCGAGATTTTCAGGAGCTAAGGAAGCTAAAATGGAGAAAAAAATCACTGGATATACCACCGTTGATATATCCCAATGGCATCGTAAAGAACATTTTGAGGCATTTCAGTCAGTTGCTCAATGTACCTATAACCAGACCGTTCAGCTGGATATTACGGCCTTTTTAAAGACCGTAAAGAAAAATAAGCACAAGTTTTATCCGGCCTTTATTCACATTCTTGCCCGCCTGATGAATGCTCATCCGGAATTTCGTATGGCAATGAAAGACGGTGAGCTGGTGATATGGGATAGTGTTCACCCTTGTTACACCGTTTTCCATGAGCAAACTGAAACGTTTTCATCGCTCTGGAGTGAATACCACGACGATTTCCGGCAGTTTCTACACATATATTCGCAAGATGTGGCGTGTTACGGTGAAAACCTGGCCTATTTCCCTAAAGGGTTTATTGAGAATATGTTTTTCGTCTCAGCCAATCCCTGGGTGAGTTTCACCAGTTTTGATTTAAACGTGGCCAATATGGACAACTTCTTCGCCCCCGTTTTCACCATGGGCAAATATTATACGCAAGGCGACAAGGTGCTGATGCCGCTGGCGATTCAGGTTCATCATGCCGTTTGTGATGGCTTCCATGTCGGCAGAATGCTTAATGAATTACAACAGTACTGCGATGAGTGGCAGGGCGGGGCGTAATTTGATATCGAGCTCGCTTGGACTCCTGTTGATAGATCCAGTAATGACCTCAGAACTCCATCTGGATTTGTTCAGAACGCTCGGTTGCCGCCGGGCGTTTTTTATTGGTGAGAATCCAAGCCTCGAGCTGTCAGACCAAGGAAGTGGATGGCCCCGTATTGCGTAATCCCTTCAGTGGTACTCCCATTGGGCAAGAGGTGGGTTTAGCGGTTAAAGATCTGGCCACAGGTCATGAAATTTATCAGTACACTGACCCAGATGGGAAGGTATTTTATGCTTCCTTTGCTGCCGCTGATGACCAAGCCACGGATTTAACCACGGCGATCGCCAATCCCACGGCCATCGATTTAATTAACGCCAGGGGATTTACGGCGGGTAGTTCCGTCACCGTATCGGGTTCCTACAGTCGGGAAGCCTTTTTTGATGGATCCATGGGTTTTTATCGACTTCTGGACGATAACGGTGCAGTGCTAGATCCCTTAACAGGTGGTGTAATCAACCCAGGACAGGTAGGTTATCAAGAAGCAGCTTTGGCAGATAGCAATCGTTTGCAAGCCACTGGCTCCACCCTAACGGCAGAAGACCTAGAAACCAGAGCATTTTCCTTCAATATTTTGGGTGGCGAGTTGTATGCGCCATTTTTAACGGTTAATGACAGTCTTTCCGGTATTAATCAGACTTATTTTGCCTTTGGGTCGGCCAACCCAGATGGCATCAGCCACAGCACAAACTTGGGACCTACTAGTAGCGGCCGCTGCAG

**DNA sequence of the chloramphenicol resistance-only plasmid for insertion into the *Synechocystis* PCC 6803 chromosome**

Key: pMB1-replicon, BioBrick prefix, *slr0168* recombination sequence, *ilvGEDA*_T, Predicted promoter and RBS, *cat* CDS (CmR), Predicted terminator, *slr0168* recombination sequence, BioBrick suffix

CCCGTAGAAAAGATCAAAGGATCTTCTTGAGATCCTTTTTTTCTGCGCGTAATCTGCTGCTTGCAAACAAAAAAACCACCGCTACCAGCGGTGGTTTGTTTGCCGGATCAAGAGCTACCAACTCTTTTTCCGAAGGTAACTGGCTTCAGCAGAGCGCAGATACCAAATACTGTCCTTCTAGTGTAGCCGTAGTTAGGCCACCACTTCAAGAACTCTGTAGCACCGCCTACATACCTCGCTCTGCTAATCCTGTTACCAGTGGCTGCTGCCAGTGGCGATAAGTCGTGTCTTACCGGGTTGGACTCAAGACGATAGTTACCGGATAAGGCGCAGCGGTCGGGCTGAACGGGGGGTTCGTGCACACAGCCCAGCTTGGAGCGAACGACCTACACCGAACTGAGATACCTACAGCGTGAGCTATGAGAAAGCGCCACGCTTCCCGAAGGGAGAAAGGCGGACAGGTATCCGGTAAGCGGCAGGGTCGGAACAGGAGAGCGCACGAGGGAGCTTCCAGGGGGAAACGCCTGGTATCTTTATAGTCCTGTCGGGTTTCGCCACCTCTGACTTGAGCGTCGATTTTTGTGATGCTCGTCAGGGGGGCGGAGCCTATGGAAAAACGCCAGCAACGCGGCCTTTTTACGGTTCCTGGCCTTTTGCTGGCCTTTTGCTCACATGAATTCGCGGCCGCTTCTAGAGACCCCTACGTGGCCGGCAATGGTCCCAAAATTGTCGCCGCTAAGTTAGACCGCTTCAGTGACCTGGGGGAAGGGGCTCCCCTCTGGTTAGCCACCAATCAAAATAACAGTGGCGGGGATTTATATGGAGACCAAGCCCAATTTCGTTTGCGAATTTACACCAGCGCCGGTTTTTCCCCCGATGGCATTGCCAGTTTACTACCCACAGAATTTGAACGGTATTTTCAACTCCAAGCGGAAGATATTACGGGACGGACAGTTATCCTAACCCAAACTGGTGTTGATTATGAAATTCCCGGCTTTGGTCTGGTGCAGGTGTTGGGGCTGGCGGATTTGGCCGGGGTTCAGGACAGCTATGACCTGACTTACATCGAAGATCATGACAACTATTACGACATTATCCTCAAAGGGGACGAAGCCGCAGTTCGCCAAATTAAGAGGGTTGCTTTGCCCTCCGAAGGGGATTATTCGGCGGTTTATAATCCCGGTGGCCCCGGCAATGATCCAGAGAATGGTCCCCCAGGGCCCTTTACTGTGTCCAGTAGTCCCCAGGTAATTAAGGTAACGGATACCATCGGCCAGCCCACCAAAGTCTCCTATGTTAGAGATCAAGCCTTAACGAACTAAGACCCCCGCACCGAAAGGTCCGGGGGTTTTTTTTGACCTTAAAAACATAACCGAGGAGCAGACACGAGTTGATCGGGCACGTAAGAGGTTCCAACTTTCACCATAATGAAATAAGATCACTACCGGGCGTATTTTTTGAGTTATCGAGATTTTCAGGAGCTAAGGAAGCTAAAATGGAGAAAAAAATCACTGGATATACCACCGTTGATATATCCCAATGGCATCGTAAAGAACATTTTGAGGCATTTCAGTCAGTTGCTCAATGTACCTATAACCAGACCGTTCAGCTGGATATTACGGCCTTTTTAAAGACCGTAAAGAAAAATAAGCACAAGTTTTATCCGGCCTTTATTCACATTCTTGCCCGCCTGATGAATGCTCATCCGGAATTTCGTATGGCAATGAAAGACGGTGAGCTGGTGATATGGGATAGTGTTCACCCTTGTTACACCGTTTTCCATGAGCAAACTGAAACGTTTTCATCGCTCTGGAGTGAATACCACGACGATTTCCGGCAGTTTCTACACATATATTCGCAAGATGTGGCGTGTTACGGTGAAAACCTGGCCTATTTCCCTAAAGGGTTTATTGAGAATATGTTTTTCGTCTCAGCCAATCCCTGGGTGAGTTTCACCAGTTTTGATTTAAACGTGGCCAATATGGACAACTTCTTCGCCCCCGTTTTCACCATGGGCAAATATTATACGCAAGGCGACAAGGTGCTGATGCCGCTGGCGATTCAGGTTCATCATGCCGTTTGTGATGGCTTCCATGTCGGCAGAATGCTTAATGAATTACAACAGTACTGCGATGAGTGGCAGGGCGGGGCGTAATTTGATATCGAGCTCGCTTGGACTCCTGTTGATAGATCCAGTAATGACCTCAGAACTCCATCTGGATTTGTTCAGAACGCTCGGTTGCCGCCGGGCGTTTTTTATTGGTGAGAATCCAAGCCTCGAGCTGTCAGACCAAGGAAGTGGATGGCCCCGTATTGCGTAATCCCTTCAGTGGTACTCCCATTGGGCAAGAGGTGGGTTTAGCGGTTAAAGATCTGGCCACAGGTCATGAAATTTATCAGTACACTGACCCAGATGGGAAGGTATTTTATGCTTCCTTTGCTGCCGCTGATGACCAAGCCACGGATTTAACCACGGCGATCGCCAATCCCACGGCCATCGATTTAATTAACGCCAGGGGATTTACGGCGGGTAGTTCCGTCACCGTATCGGGTTCCTACAGTCGGGAAGCCTTTTTTGATGGATCCATGGGTTTTTATCGACTTCTGGACGATAACGGTGCAGTGCTAGATCCCTTAACAGGTGGTGTAATCAACCCAGGACAGGTAGGTTATCAAGAAGCAGCTTTGGCAGATAGCAATCGTTTGCAAGCCACTGGCTCCACCCTAACGGCAGAAGACCTAGAAACCAGAGCATTTTCCTTCAATATTTTGGGTGGCGAGTTGTATGCGCCATTTTTAACGGTTAATGACAGTCTTTCCGGTATTAATCAGACTTATTTTGCCTTTGGGTCGGCCAACCCAGATGGCATCAGCCACAGCACAAACTTGGGACCTACTAGTAGCGGCCGCTGCAG
